# Catalytic Dual‐Mode Immunotherapy: Anisotropic AuPt Heterostructure Decorated with Starry Pt Nanoclusters for Robust Cancer Photometalloimmunotherapy

**DOI:** 10.1002/advs.202403116

**Published:** 2024-05-30

**Authors:** Wei Bian, Xi Hu, Ruixue Xiao, Rui Yao, Bo Zhang, Mingjian Zhu, Tianqi Liu, Yamin Liu, Jing Li, Peihua Lin, An Xie, Fangyuan Li, Daishun Ling

**Affiliations:** ^1^ Department of Breast Surgery First Hospital of Shanxi Medical University Taiyuan 030001 China; ^2^ Key Laboratory of Cellular Physiology at Shanxi Medical University Ministry of Education Taiyuan 030000 China; ^3^ School of Pharmacy Anhui University of Chinese Medicine Hefei 230012 China; ^4^ Frontiers Science Center for Transformative Molecules School of Chemistry and Chemical Engineering School of Biomedical Engineering National Center for Translational Medicine Shanghai Jiao Tong University Shanghai 200240 China; ^5^ WLA Laboratories Shanghai 201203 China; ^6^ Institute of Pharmaceutics Hangzhou Institute of Innovative Medicine College of Pharmaceutical Sciences Zhejiang University Hangzhou 310058 China; ^7^ Songjiang Institute and Songjiang Hospital Shanghai Key Laboratory of Emotions and Affective Disorders Shanghai Jiao Tong University School of Medicine Shanghai 200025 China; ^8^ Key Laboratory of Precision Diagnosis and Treatment for Hepatobiliary and Pancreatic Tumor of Zhejiang Province Hangzhou 310009 China

**Keywords:** cancer photometalloimmunotherapy, dumbbell‐like heterostructures, immunogenic cell death, ion release, platinum nanoclusters

## Abstract

To overcome current limitations in photoimmunotherapy, such as insufficient tumor antigen generation and a subdued immune response, a novel photo‐/metallo dual‐mode immunotherapeutic agent (PMIA) is introduced for potent near‐infrared (NIR) light‐triggered cancer therapy. PMIA features a dumbbell‐like AuPt heterostructure decorated with starry Pt nanoclusters, meticulously engineered for enhancing plasmonic catalysis through multi‐dimensional regulation of Pt growth on Au nanorods. Under NIR laser exposure, end‐tipped Pt nanoclusters induce efficient electron‐hole spatial separation along the longitudinal axis, resulting in radial and axial electron distribution polarization, conferring unique anisotropic properties to PMIA. Additionally, starry Pt nanoclusters on the sides of Au nanorods augment the local electron enrichment field. Validated through finite‐difference time‐domain analysis and Raman scattering, this configuration fosters local electron enrichment, facilitating robust reactive oxygen species generation for potent photoimmunotherapy. Moreover, Pt nanoclusters facilitate Pt^2+^ ion release, instigating intranuclear DNA damage and inducing synergistic immunogenic cell death (ICD) for metalloimmunotherapy. Consequently, PMIA elicits abundant danger‐associated molecular patterns, promotes T cell infiltration, and triggers systemic immune responses, effectively treating primary and distant tumors, inhibiting metastasis in vivo. This study unveils a pioneering dual‐mode ICD amplification strategy driven by NIR light, synergistically integrating photoimmunotherapy and metalloimmunotherapy, culminating in potent cancer photometalloimmunotherapy.

## Graphical abstract

A novel photo‐/metallo dual‐mode immunotherapeutic agent (PMIA) is presented characterized by a unique dumbbell‐like AuPt heterostructure decorated with starry Pt nanoclusters. This design is meticulously crafted through precise orchestration of heteroepitaxial multi‐site growth on Au nanorods, seamlessly integrating photo‐ and metal ion‐mediated immunogenic cell death. The PMIA manifests a robust and dual immunotherapeutic efficacy, heralding a new paradigm termed as photometalloimmunotherapy.

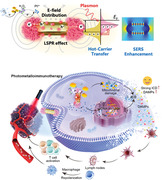

## 1 Introduction

Phototherapy, encompassing photothermal therapy (PTT),^[^ ^1^ ^]^ photodynamic therapy (PDT),^[^ ^2^ ^]^ and photocatalytic therapy (PCT),^[^ ^3^ ^]^ has emerged as a promising approach for precise tumor therapeutics, owing to its remarkable spatiotemporal controllability in a noninvasive manner.^[^ ^4^ ^]^ Upon light activation, phototherapeutic agents can induce local heat and/or generate reactive oxygen species (ROS) within tumors, thereby triggering immunogenic cell death (ICD) and the release of damage‐associated molecular patterns (DAMPs) from apoptotic or necrotic cell debris.^[^ ^5^ ^]^ DAMPs including calreticulin (CRT), high mobility group box 1 (HMGB1), and adenosine triphosphate (ATP), act as signals to recruit antigen‐presenting cells (APCs) such as dendritic cells (DCs), initiating tumor‐specific immune responses.^[^ ^6^ ^]^ However, despite these promising outcomes, conventional phototherapy often falls short in generating sufficient DAMPs and is impeded by power attenuation in thick biological tissues, leading to inadequate DC maturation and immune response.^[^ ^7^ ^]^

The evolving field of immunology has underscored the vital roles of metal ions in modulating the tumor microenvironment (TME) and immune cell function, garnering significant attention for their potential to enhance antitumor immune responses.^[^ ^8^ ^]^ For instance, zinc (Zn^2+^) and calcium (Ca^2+^) exhibit the ability to modulate T cell activation,^[^ ^9^ ^]^ while manganese (Mn^2+^) demonstrates the capability to activate the cGAS‐STING pathway.^[^ ^10^ ^]^ Platinum (Pt)‐based nanoagents have recently gained prominence in tumor therapy.^[^ ^11^ ^]^ Remarkably, finely tuned Pt‐based metallic nanoparticles can undergo facile oxidation, releasing Pt^2+^ ions that can enter the nucleus and induce DNA damage,^[^ ^12^ ^]^ consequently, triggering cell apoptosis and augmenting ICD.^[^ ^13^ ^]^

Dual noble metal heterostructures (HSs), exemplified by gold‐platinum (AuPt) HSs, equipped both plasmon‐mediated catalytic properties and free metal ion‐driven processes.^[^ ^14^ ^]^ By controllably engineering anisotropic architectures, AuPt HSs exhibit promising phototherapeutic effect under near‐infrared (NIR) laser irradiation. For example, in contrast to Au@Pt core‐shell nanorods (NRs), dumbbell‐shaped AuPt HSs could achieve efficient electron‐hole spatial separation, leading to enhanced ROS production for tumor phototherapy.^[^ ^15^ ^]^ Furthermore, Janus AuPt nanomotors can selectively release cytotoxic Pt^2+^ ions into the nucleus, thereby causing DNA damage and cell apoptosis.^[^ ^16^ ^]^ In light of these considerations, there is a critical need to develop a photoimmunotherapeutic agent that integrates metal ion‐mediated ICD to overcome the limitations of current photoimmunotherapy. Such an agent has the potential to elicit a robust photo‐/metallo dual immunotherapeutic effect, presenting an unexplored avenue in cancer therapy.

**Figure 1:**
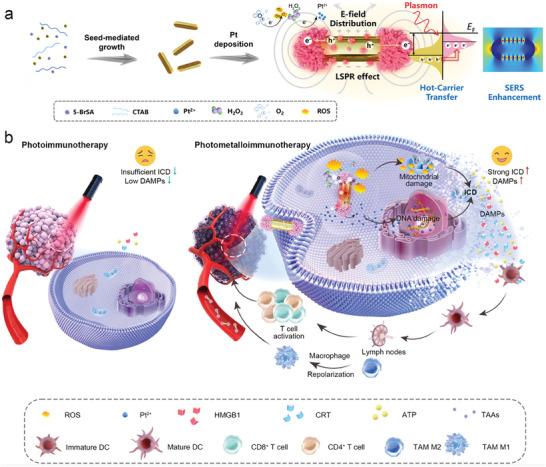
Designing the Photo‐/Metallo Dual‐Mode Immunotherapeutic Agent (PMIA) for robust near‐infrared (NIR) photometalloimmunotherapy. a) Schematic illustration of PMIA design through multi‐dimensional regulation of heteroepitaxial multi‐site Pt growth on Au NRs. PMIA enhances efficient electron‐hole spatial separation and intensifies the local electron enrichment field under NIR laser irradiation, thereby promoting ROS generation and Pt^2+^ ions release. b) PMIA induces intranuclear DNA damage and amplifies immunogenic cell death (ICD), eliciting robust antitumor immune responses. As well as, PMIA exhibits resilience against NIR attenuation, synergizing the strengths of both photoimmunotherapy and metalloimmunotherapy.

In this study, we report a photo‐/metallo dual‐mode immunotherapeutic agent (PMIA) by decorating anisotropic dumbbell‐like AuPt HSs with starry Pt nanoclusters, achieved through precise tuning of optimal heteroepitaxial multi‐sites on Au NRs. Under NIR laser irradiation, end‐tipped Pt nanoclusters induce efficient electron‐hole spatial separation along the NRs’ longitudinal axis, leading to radial and axial electron distribution polarization for the anisotropic PMIA. Additionally, Pt nanoclusters on the side of Au nanorods create an intensified local electron enrichment field. Arising from this ingenious spatial configuration, PMIA enhances plasmon‐mediated catalytic reactions for abundant ROS generation and provides excess active sites, remarkably facilitating Pt^2+^ ions release (**Figure** **1**a). In accordance with expectation, PMIA induces significant intranuclear DNA damage and amplifies ICD during plasmonic catalysis under low‐power NIR irradiation (0.05 W cm^−2^), demonstrating resilience against attenuation as light penetrates deep into tissues. Moreover, PMIA promotes effector T cell infiltration into tumors and activates systemic immune responses, effectively treating both primary and distant tumors, and inhibiting metastasis in vivo (Figure 1b). Our study presents PMIA as a promising photo‐/metallo dual‐mode immunotherapeutic agent for robust cancer photometalloimmunotherapy, offering a novel therapeutic paradigm.

## 2 Results and Discussion

**Figure 2:**
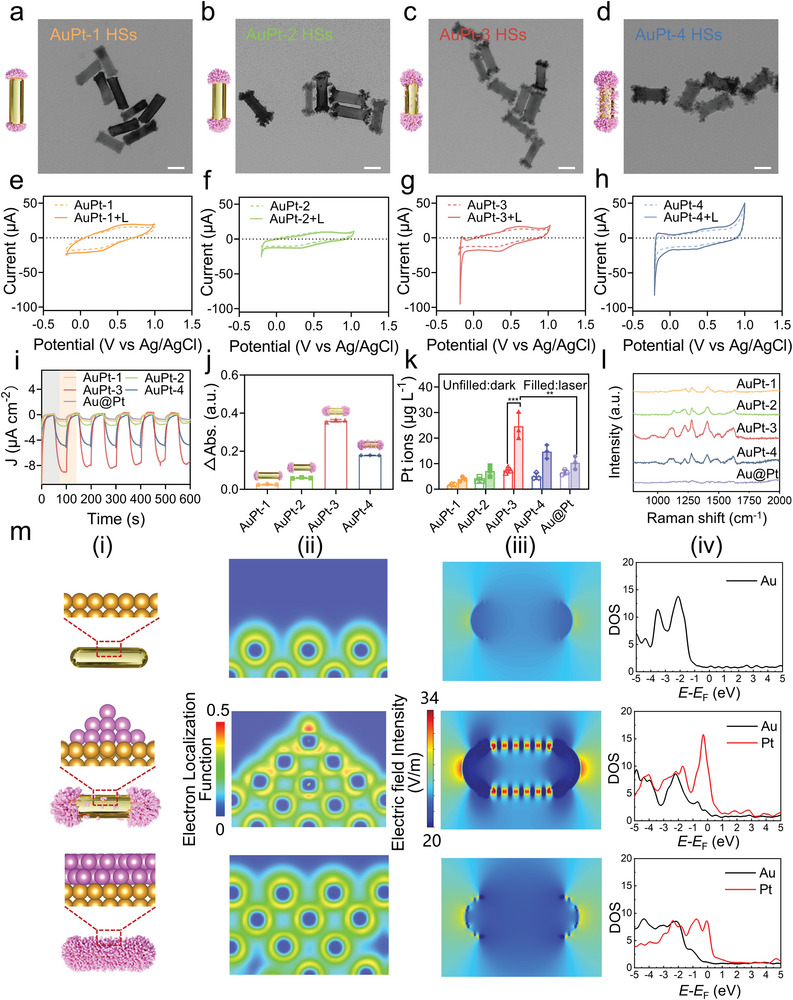
Fine‐tuning and characterization of anisotropic dumbbell‐like AuPt HSs for optimized PMIA. a–d) Transmission electron microscopy (TEM) images of AuPt‐1 HSs (a), AuPt‐2 HSs (b), AuPt‐3 HSs (c), and AuPt‐4 HSs (d), respectively. Scale bar, 25 nm. e–h) Cyclic voltammogram (CV) curves of AuPt‐1 HSs (e), AuPt‐2 HSs (f), AuPt‐3 HSs (g), and AuPt‐4 HSs (h) with (solid line) or without (dotted line) laser irradiation. i) Photocurrent curves of AuPt HSs and Au@Pt NRs under 808 nm laser irradiation. j) The UV–vis absorption increment of TMB oxidation product at 652 nm in the presence of AuPt HSs and H_2_O_2_ after 808 nm laser irradiation. Data are presented as mean ±  SD (*n*  =  3/group). k) The accumulated Pt^2+^ ions release from AuPt HSs and Au@Pt NRs in PBS (pH 5.5, 10 mm) under 808 nm laser irradiation (0.5 W cm^−2^, 5 min). Data are presented as mean ± SD (*n*  =  3/group), ^**^ *p* < 0.01, ^***^ *p* < 0.001. l) SERS spectra of Au@Pt NRs and a series of AuPt HSs. m) Density functional theory (DFT) and finite‐difference time‐domain (FDTD) analysis to investigate the electron enrichment and local electrical field intensity of different forms of Pt deposition, including no Pt (top), Pt nanoclusters (middle) and Pt shells (bottom) on Au surface. i) schematic diagram; ii) electron localization function (ELF) analysis; iii) spatial distribution of enhanced electric field; iv) density of states (DOS) analysis. The numbers of Au or Pt atoms were set to be the same.

Au NRs were synthesized via a seed‐mediated growth procedure,^[^ ^17^ ^]^ resulting in uniform sizes of 40 ± 5 nm in length and 12 ± 2 nm in diameter (Figure S1, Supporting Information). Anisotropic dumbbell‐like AuPt HSs can be obtained by selectively depositing multi‐site Pt growth, assisted by 5‐bromosalicylic acid to block specific sides of Au NRs.^[^ ^18^ ^]^ By adjusting the amount of Pt precursors in different molar ratios (Pt: Au = 1:10, 1:5, 1:3.3, and 1:2.5), a series of AuPt HSs (termed as AuPt‐1 HSs, AuPt‐2 HSs, AuPt‐3 HSs, and AuPt‐4 HSs, respectively) with various Pt deposition degree and locations were fabricated (**Figure** **2**a–d). Starry Pt nanoclusters were found grown on the middle surface of AuPt‐3 HSs, uniformly covering the Au NRs (termed as Au@Pt NRs; Figure S2, Supporting Information) when further raising Pt: Au molar ratios. Inductively coupled plasma mass spectrometry (ICP‐MS) analysis indicated AuPt‐1 HSs, AuPt‐2 HSs, AuPt‐3 HSs, AuPt‐4 HSs, and Au@Pt NRs possessed Pt: Au molar ratio at 1: 20, 1:8, 1:5, 1:3, and 1:1, respectively. After surface modification by methoxy poly (ethylene glycol)−5000‐thiol (mPEG_5k_‐SH), the electrochemical characteristics of AuPt HSs, Au@Pt NRs, and Au NRs were evaluated using a three‐electrode system (Figure 2e–h; Figures S3 and S4, Supporting Information).^[^ ^19^ ^]^ The cyclic voltammogram (CV) curves of AuPt‐3 HSs exhibited a much more distinct redox peak when exposed to 808 nm NIR laser irradiation compared to other counterparts, indicating the most drastic plasmon‐excitation induced hot electron transfer inside AuPt‐3 HSs under laser irradiation.^[^ ^20^ ^]^ Furthermore, AuPt‐3 HSs performed a notably enhanced photocurrent upon 808 nm laser irradiation, much higher than AuPt‐4 HSs, whereas AuPt‐1 HSs, AuPt‐2 HSs, and Au@Pt NRs yielded relatively weak responses (Figure 2i). This suggests that Pt deposition uniquely promotes the separation of photo‐induced electrons and holes.^[^ ^21^ ^]^ Moreover, the localized surface plasmon resonance (LSPR) band shift of AuPt HSs ranged from 740 to 860 nm with increasing Pt precursors (Figure S5, Supporting Information), indicating the role of Pt deposition (on both ends or side of the Au NRs) in adjusting the optical properties.^[^ ^22^ ^]^ Besides, Figure S6 (Supporting Information) shows that these AuPt HSs exhibited the comparable photothermal activities.

To assess the catalytic efficacy of various AuPt HSs in practical catalytic reactions, we conducted investigations using 3,3′,5,5′‐tetramethylbenzidine (TMB) as the substrate in the presence of H_2_O_2_.^[^ ^23^ ^]^ The absorbance values of these AuPt HSs were modulated to be consistent at 808 nm by adjusting concentrations to eliminate the photo‐absorption difference of AuPt HSs. As shown in Figure S7a (Supporting Information), the absorbance increment of AuPt‐3 HSs under dark conditions was 1.61‐ and 3.11‐fold higher than that of AuPt‐4 and AuPt‐2 HSs after incubation for 30 min, respectively. Notably, the TMB oxidation of AuPt‐3 HSs raised by 2.05‐fold under 808 nm laser irradiation (0.5 W cm^−2^, 5 min) for 30 min (Figure S7b, Supporting Information) and showed 1.79‐fold enhancement compared to that of AuPt‐4 HSs + laser (L) group (Figure 2j).^[^ ^24^ ^]^ As expected, the anisotropic AuPt HSs, particularly the Pt nanoclusters deposited at both ends of Au NRs, facilitate the generation of efficient electron‐hole pairs, ensuring radial electron polarization for sustained progress of catalytic reactions. In further investigation, we evaluated the release behavior of Pt^2+^ ions from both AuPt HSs and Au@Pt NRs with or without 808 nm laser irradiation. Remarkably, AuPt‐3 HSs demonstrated a superior NIR light‐triggered Pt^2+^ release profile, ≈2.91 times greater than that observed from Au@Pt NRs after 808 nm irradiation at 0.5 W cm^−2^ for 5 min, as measured by 24 h (Figure 2k). We further compared the desorption energy of Pt ions on AuPt‐3 HSs and Au@Pt NRs. As revealed in Figure S8 (Supporting Information), Pt ions on Pt nanoclusters occupied a lower desorption energy barrier (5.72 eV) compared with the that on Pt shell (10.94 eV), underscoring the crucial role of Pt nanoclusters in Pt ions releasing. Additionally, we performed surface enhancement of Raman scattering (SERS) detection on a series of AuPt HSs. The SERS signal of AuPt‐3 HSs, loaded with starry Pt nanoclusters, exhibited significant enhancement compared to Au@Pt NRs and other AuPt HSs counterparts (Figure 2l), demonstrating the local electric field enhancement induced by deposited Pt nanoclusters.^[^ ^25^ ^]^

Next, density functional theory (DFT) and finite‐difference time‐domain (FDTD) analysis were conducted to explore the electron enrichment and local electric field intensity resulting from various forms of Pt on the Au surface (Figure 2m).^[^ ^26^ ^]^ The electron localization function (ELF) results revealed distinct behaviors for Pt nanoclusters and shells compared to the absence of Pt on the Au surface. Specifically, Pt nanoclusters led to pronounced electron enrichment at the Au surface, concentrating electrons predominantly on the top Pt atom. In stark contrast, the formation of a homogeneous Pt shell on the Au surface did not exhibit noticeable electron enrichment (Figure 2m‐i,ii). Furthermore, FDTD analysis showed that Pt nanoclusters loaded on the AuPt HSs side surface can produce high local electric field intensity distribution on both ends and side surface of dumbbell‐like AuPt HSs, which was consistent with the above rules and highlight the critical role of Pt nanoclusters in achieving enhanced SERS performance within AuPt‐3 HSs, paving the way for novel applications in catalysis and sensing (Figure 2m‐iii; Figure S9, Supporting Information).^[^ ^27^ ^]^ Furthermore, the Pt growth on the Au surface adjusted the density of states (DOS) closer to the Fermi level (Figure 2m‐iv; Figure S10, Supporting Information), facilitating easier electron transfer across the energy barrier and thereby enhancing the electronic activity of the surface.

Under NIR laser irradiation, Pt nanoclusters exhibit dual effects within AuPt‐3 HSs. First, Pt nanoclusters deposited at the end sites induce radial electron distribution polarization, enhancing the efficiency of carrier production. Second, Pt nanoclusters loaded on the sides of Au NRs significantly boost the local electric field due to SERS enhancement, which not only promotes the excitation and transition of photons, leading to the formation of electron‐hole pairs, but also reduces the reaction barrier for catalytic substrates near the “hotspots” of PMIA.^[^ ^28^ ^]^ Optimal deposition numbers and well‐arranged Pt nanoclusters result in an abundance of active sites and facilitated efficient migration pathways for the release of Pt^2+^.^[^ ^29^ ^]^

**Figure 3:**
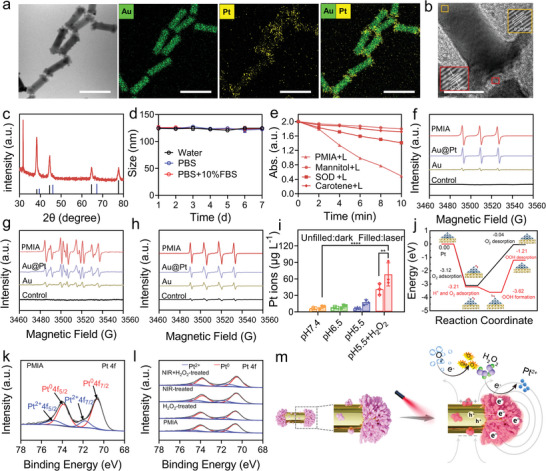
Characterization and catalytic performance of anisotropic PMIA. a) High‐angle annular dark‐field scanning transmission electron microscope (HAADF‐STEM) image and elemental mapping of PMIA. Scale bar, 50 nm. b) HRTEM image of PMIA. Scale bar, 10 nm. c) XRD pattern with distinct peaks corresponding to Au (JCPDS card no. 65–2870) and Pt (JCPDS card no. 65–2868). d) Size distribution of PEGylated PMIA after storage in distilled water, PBS, and 10% FBS/PBS for 7 days. Data presented as mean ± SD (*n* = 3/group). e) Time‐course absorbance of DPBF absorbance at 410 nm with different ROS scavengers in the presence of PMIA under 808 nm laser irradiation. f–h) ESR spectra of TEMP‐^1^O_2_ spin adducts (f), BMPO‐•O_2_^−^ spin adducts (g), and DMPO‐•OH spin adducts (h) generated by PMIA, Au NRs, and Au@Pt NRs under 808 nm laser irradiation (0.5 W cm^−2^, 5 min). i) Accumulated Pt^2+^ ions release from PMIA in various conditions with 808 nm laser irradiation (0.5 W cm^−2^, 5 min). Data presented as mean ± SD (*n* = 3/group), ^**^ *p* < 0.01, ^****^ *p* < 0.0001. j) The energy barrier of ROS formation by oxygen molecule reacting on the surface of PMIA with or without hydrogen ion by using DFT method. k) XPS spectra of Pt 4f for PMIA. l) XPS spectra of Pt 4f for PMIA with different treatments. m) Schematic illustration depicting the tuning of hot carriers‐mediated plasmonic catalysis for ROS generation and Pt^2+^ ion releasing.

We conducted an in‐depth exploration into the structural excellence of AuPt‐3 HSs, referred to as PMIA henceforth. First, energy‐dispersive X‐ray spectroscopic (EDS) mapping verified multi‐site Pt deposition on both ends and along the length of the Au NRs, confirming successful decoration of anisotropic dumbbell‐like AuPt HSs with starry Pt nanoclusters (**Figure** **3**a). High‐resolution transmission electron microscope (HRTEM) image revealed distinct and continuous lattice fringes of PMIA (Figure 3b), corresponding to the crystal planes of Au (200) and Pt (111) in X‐ray diffraction (XRD) patterns (Figure 3c), indicative of an epitaxial growth of Pt on Au. Moreover, PMIA exhibited a negative charge (Figure S11, Supporting Information) and excellent stability post‐PEGylation, as confirmed by dynamic light scattering (DLS) measurements (Figure 3d). Benefiting from its unique electron‐hole separation efficiency, PMIA demonstrated remarkable reaction rates towards 1,3‐diphenylisobenzofuran (DPBF) under 808 nm laser irradiation. This reaction could be significantly suppressed by exposure to carotene (a ^1^O_2_ quencher), mannitol (a •OH quencher), and superoxide dismutase (SOD, a •O_2_^−^ quencher) by 92%, 91%, and 67%, respectively (Figure 3e).^[^ ^30^ ^]^ Furthermore, electron spin resonance (ESR) coupled with trapping agents consistently confirmed the generation of plasmon‐excited ROS,^[^ ^31^ ^]^ specifically •OH, ^1^O_2_, and •O_2_^−^ (Figure 3f–h), indicating photon‐enhanced peroxidase‐like activities. Notably, cumulative laser‐induced Pt^2+^ release from PMIA significantly increased when treated with pH 5.5 PBS and/or H_2_O_2_ (10 mm) (Figure 3i), highlighting the pH and H_2_O_2_ dependency of Pt^2+^ release from PMIA. Furthermore, we compared the energy barrier of ROS formation and Pt^2+^ release on the surface of PMIA with and without hydrogen (H^+^) ions using DFT calculations. These results indicate that higher concentrations of H^+^ ions under acidic conditions induce lower reaction energies and energy barriers during catalytic ROS generation. This phenomenon enhances ROS generation on the surface of Pt nanoclusters, thereby promoting Pt^2+^ ion release (Figure 3j).

This finding lays the groundwork for tumor microenvironment‐responsive DNA damage and ICD activation. X‐ray photoelectron spectroscopy (XPS) spectra revealed distinct peaks at 70.67 and 74.02 eV, which could be corresponding to Pt^0^ and Pt^2+^, respectively (Figure 3k).^[^ ^32^ ^]^ Importantly, the Pt 4f_7/2_ binding energy of PMIA exhibited a negative shift of ≈0.10 eV after 808 nm laser irradiation (Figure 3l), indicating a strong interaction between Au and Pt.^[^ ^33^ ^]^ Additionally, the proportion of Pt^2+^ sharply increased upon treatment with H_2_O_2_ and/or laser irradiation (Figure 3l), consistent with the Pt^2+^ leaching results (Figure 3i).^[^ ^32^ ^]^ Collectively, under NIR laser irradiation, PMIA, featuring a preponderant heteroepitaxial Pt nanocluster deposition, achieves a significant improvement in ROS generation efficiency and Pt^2+^ ion leaching (Figure 3m).

**Figure 4:**
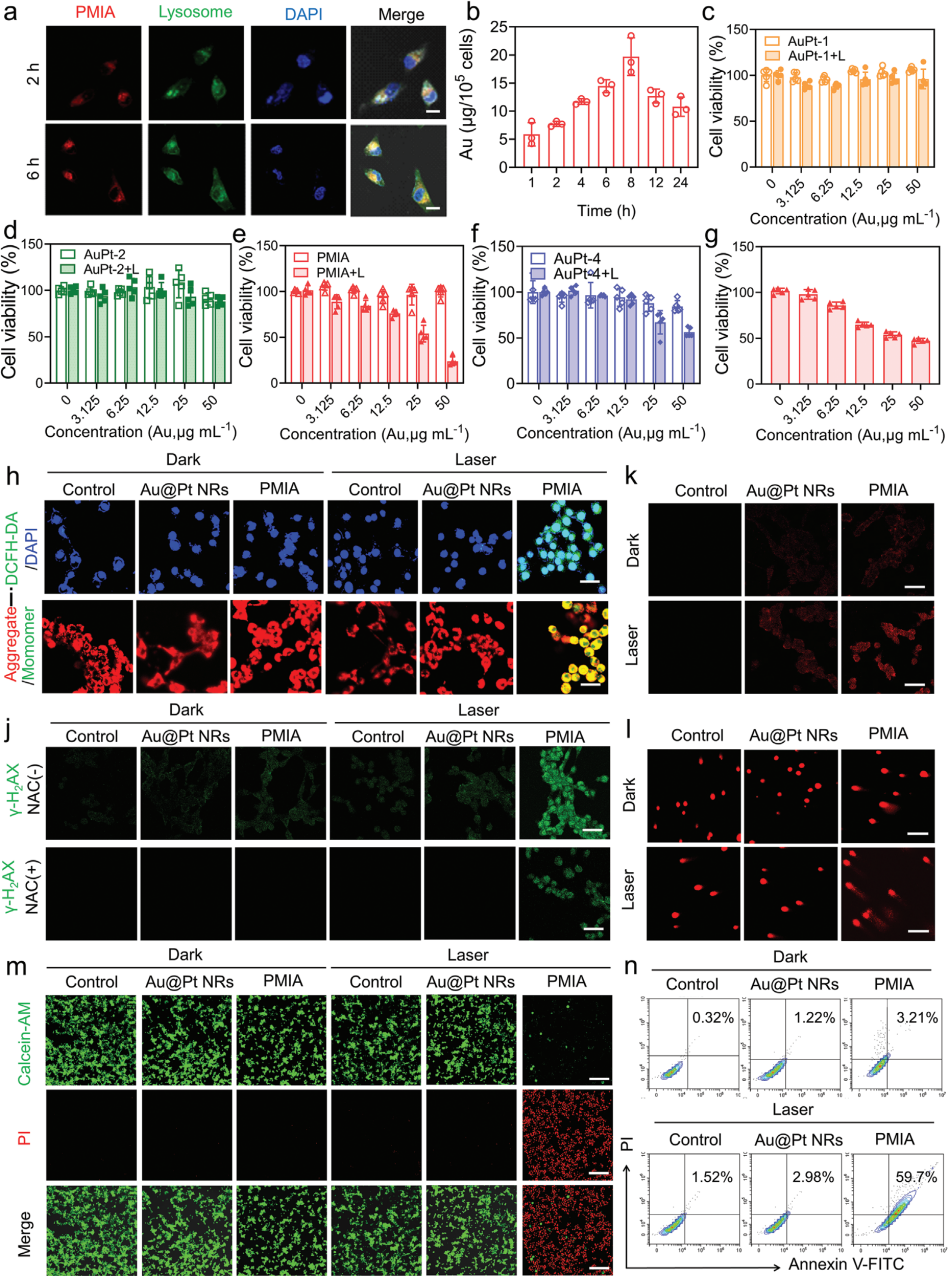
Plasmonic catalysis‐mediated cell death induced by PMIA. a) CLSM images of 4T1 cells after incubation with RITC‐labeled PMIA for different times. Scale bar, 25 µm. b) The concentration of Au in 4T1 cells after incubation with PMIA at different time points. Data are presented as mean ± SD (*n* = 3/group). c–f) Viabilities of 4T1 cells after incubation with AuPt‐1 HSs (c), AuPt‐2 HSs (d), PMIA (e), and AuPt‐4 HSs (f) without and with 808 nm laser irradiation (0.5 W cm^−2^). Data are presented as mean ± SD (*n* = 5/group). g) Photocytotoxicity of PMIA at different [Au] concentrations (0.05 W cm^−2^). Data are presented as mean ± SD (*n* = 5/group). h) CLSM images of DCFH‐DA stained 4T1 tumor cells after different treatments without or with 808 nm laser irradiation (0.05 W cm^−2^). Scale bar, 40 µm. i) CLSM images of JC‐1 stained 4T1 tumor cells after different treatments without or with 808 nm laser irradiation (0.05 W cm^−2^). The mitochondrial damage is indicated by the green fluorescent signals of JC‐1 monomers. Scale bar, 30 µm. j) Immunofluorescence staining of the γ‐H2AX in 4T1 tumor cells after different treatments without or with 808 nm laser irradiation (0.05 W cm^−2^) or NAC. Scale bar, 40 µm. k) Immunofluorescence staining of the Pt‐DNA adducts in 4T1 tumor cells after different treatments without or with 808 nm laser irradiation (0.05 W cm^−2^). Scale bar, 40 µm. l) The comet assay of 4T1 tumor cells after different treatments without or with 808 nm laser irradiation. Scale bar, 160 µm. m) CLSM images of Calcein‐AM/PI stained 4T1 tumor cells after different treatments without or with 808 nm laser irradiation. Scale bar, 200 µm. n) Flow cytometry analysis of cell apoptosis in 4T1 tumor cells after different treatments with or without 808 nm laser irradiation (0.05 W cm^−2^).

The plasmonic photocatalytic performance of AuPt HSs was investigated at the cellular level. Both confocal laser scanning microscopy (CLSM) images (**Figure** **4**a) and cellular Au concentration measured by inductively coupled plasma mass spectrometry (ICP‐MS) (Figure 4b), confirmed that AuPt HSs can be internalized into 4T1 tumor cells in a time‐dependent manner. Notably, in contrast to AuPt HSs and Au@Pt NRs with the negligible cytotoxicity (Figure 4c–f; Figure S12, Supporting Information), the photocytotoxicity of PMIA surpassed other AuPt HSs counterparts, and remained relatively constant under upon low‐power NIR laser irradiation (0.05 W cm^−2^) (Figure 4g) or in an ice condition (Figure S13, Supporting Information). This consistency aligned with their superior plasmonic catalytic performance. Additionally, PMIA + L‐treated groups exhibited elevated ROS generation (Figure 4h) and severe mitochondrial damage (Figure 4i) in 4T1 tumor cells, which is attributed to the spatially separated structure resulting from proper anisotropic deposition of heterometals (Pt nanoclusters) at both ends and side, facilitating efficient separation of hot electrons and holes, supporting electron enrichment for local E‐field intensity.

Motivated by the photon‐driven release of Pt^2+^ ions, we conducted a detailed investigation into the effects of PMIA on DNA damage. In contrast to Au@Pt NRs, PMIA induced substantial DNA damage (Figure 4j) through oxidative cleavage of DNA strands,^[^ ^12^ ^]^ which, interestingly, is partially mitigated in presence of N‐acetylcysteine (NAC) as an antioxidant‐scavenging ROS (Figure 4j).^[^ ^12^ ^]^ Our findings demonstrate that PMIA significantly induces DNA damage under NIR irradiation. Moreover, PMIA formed persistent Pt‐DNA adducts that remain unremoved (Figure 4k), likely owing to their ability to disrupt DNA structures through in situ ROS generation and Pt^2+^ ions release. Additionally, the comet assay revealed longer comet tails in PMIA + L‐treated cells compared to other groups (Figure 4l), indicating more severe DNA damage. The efficacy of PMIA in photocatalytic therapy against 4T1 cells was further validated using the Calcein‐AM/PI co‐staining assay (Figure 4m). Compared to the control groups (L, Au@Pt NRs + L, and PMIA alone), 4T1 cells treated with PMIA followed by NIR irradiation exhibited a larger area of strong red fluorescence, indicating dying cells, along with reduced green fluorescence representing living cells. These results collectively confirm the significant tumor inhibitory effect of the PMIA + L group. Furthermore, upon NIR irradiation, PMIA markedly enhances the apoptosis rate of tumor cells (Figure 4n). Collectively, PMIA facilitates substantial ROS generation and Pt^2+^ ions release under NIR light irradiation, leading to intranuclear DNA damage and efficient tumor cell killing, thereby enhancing the overall phototherapeutic effect.

**Figure 5:**
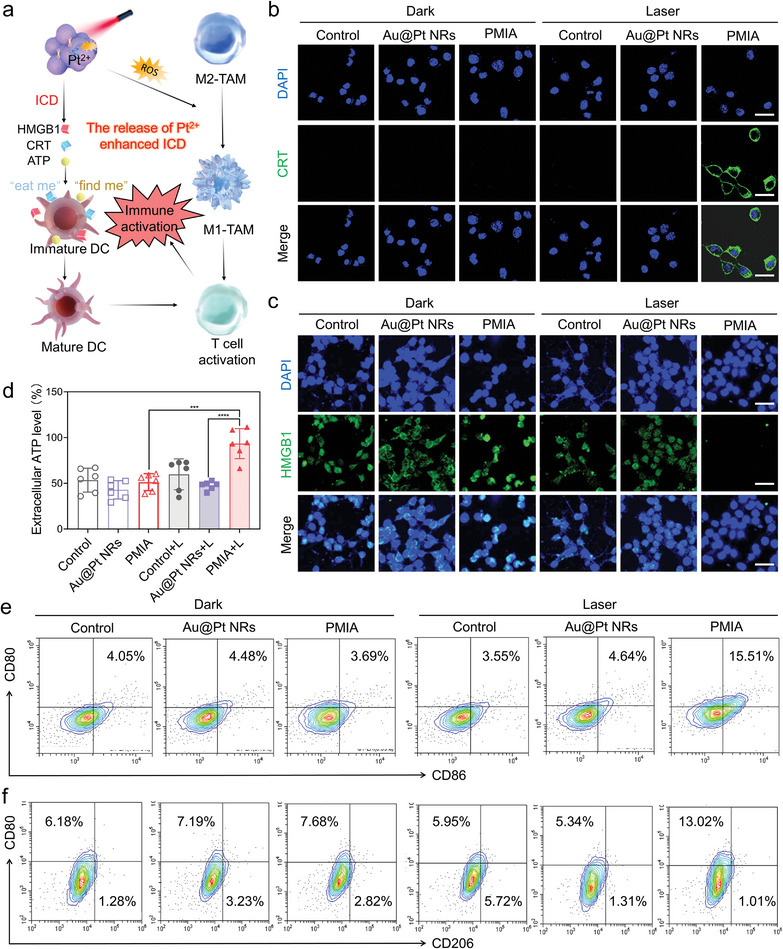
NIR light‐triggered dual‐mode ICD amplification by PMIA. a) Schematic illustration of PMIA eliciting dual‐mode enhanced ICD and immune response activation. b,c) CLSM images of CRT (b) and HMGB1 (c) in 4T1 tumor cells after different treatments with or without 808 nm laser irradiation (0.05 W cm^−2^). Scale bar, 50 µm. d) Relative ATP release level of 4T1 tumor cells after different treatments. Data are presented as mean ± SD (*n*  =  6/group), ^***^ *p* < 0.001, ^****^ *p* < 0.0001. e) The proportion of DC maturation (stained with CD80^+^CD86^+^) after different treatments with or without 808 nm laser irradiation (0.05 W cm^−2^) detected by flow cytometry. f) The proportion of M1‐phenotype macrophages (stained with CD80^+^) and M2‐phenotype macrophages (stained with CD206^+^) after different treatments with or without 808 nm laser irradiation (0.05 W cm^−2^) detected by flow cytometry.

Then, we investigated whether PMIA + L‐triggered ROS and Pt^2+^ ions would induce ICD for the activation of an immune response (**Figure** **5**a). In the context of ICD induced by phototherapy, key DAMPs molecules, including CRT, HMGB1, and ATP play pivotal roles. We evaluated CRT expression in 4T1 cells treated with Au@Pt NRs and PMIA under both laser and non‐laser conditions using CLSM. Remarkably, we observed a significant increase in CRT expression on the surface of PMIA‐treated 4T1 cells under laser irradiation (Figure 5b). Similarly, PMIA + L treatment led to a notable elevation in extracellular HMGB1 (Figure 5c) and extracellular ATP (Figure 5d) levels compared to other treatments. Furthermore, we investigated DCs, a crucial type of APCs responsible for capturing and presenting tumor‐associated antigens (TAAs) to activate T cells. After co‐incubating DCs with 4T1 cells under different treatments, PMIA + L treatment boosted the CD80^+^CD86^+^DC population to 15.51%, indicating the enhanced DC maturation (Figure 5e). This maturation could be attributed to the increased release of DAMPs from dying cancer cells.^[^ ^34^ ^]^ Macrophages play a pivotal role in antitumor immunotherapy, and their polarization from the M2‐phenotype (tumor‐associated macrophages, TAMs) to the M1‐phenotype significantly enhances immunological efficacy.^[^ ^35^ ^]^ PMIA + L‐treated group exhibited a remarkable upregulation of M1‐phenotype macrophages (CD80^+^) and a downregulation of M2‐phenotype macrophages (CD206^+^) (Figure 5f), indicating an effective repolarization of TAMs into M1‐phenotype macrophages.^[^ ^8a^ ^]^ Consequently, it is speculated that upon NIR irradiation, PMIA can effectively induce ICD and facilitate M2‐ to M1‐type TAM repolarization, thereby potentiating antitumor immunity in breast cancer for highly efficient photometalloimmunotherapy.

**Figure 6:**
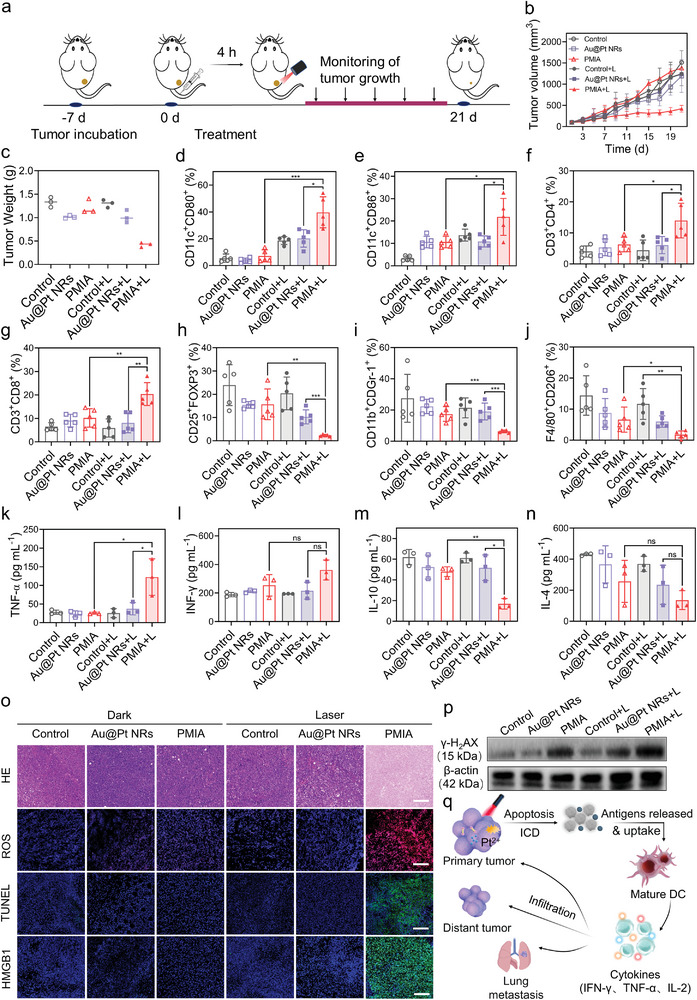
Anisotropic dumbbell‐like PMIA modulate TME to potentiate in situ tumor photometalloimmunotherapy. a) Treatment schedule of plasmonic catalysis‐driven TME modulation for photometalloimmunotherapy in 4T1‐tumor‐bearing BALB/c mice. b,c) 4T1‐tumor growth curves (b) and tumor weights (c) of mice treated with different groups. Data are presented as mean ± SD (*n* = 3/group). d,e) The quantitative analysis of matured DCs (CD11c^+^CD80^+^ and CD11c^+^CD86^+^) in lymph nodes after different treatments detected by flow cytometry. Data are presented as mean ± SD (*n* = 5/group), ^*^ *p* < 0.05, ^***^ *p* < 0.001. f,g) The proportion of the CD3^+^CD4^+^T cells (f) and CD3^+^CD8^+^T cells (g) in tumor tissues after different treatments by flow cytometry. Data are presented as mean ± SD (*n* = 5/group), ^*^ *p* < 0.05, ^**^ *p* < 0.01. h) The proportion of Tregs (stained with CD3^+^CD4^+^Foxp3^+^) in tumor tissues after different treatments by flow cytometry. Data are presented as mean ±  SD (*n* = 5/group), ^**^ *p* < 0.01, ^***^ *p* < 0.001. i) The proportion of the MDSC cells (stained with CD3^+^CD11b^+^CDGr‐1^+^) in tumor tissues after different treatments by flow cytometry. Data are presented as mean ± SD (*n* = 5/group), ^***^ *p* < 0.001. j) The proportion of M2‐TAM (stained with F4/80^+^CD206^+^) in tumor tissues after different treatments by flow cytometry. Data are presented as mean ± SD (*n* = 5/group), ^*^ *p* < 0.05, ^**^ *p* < 0.01. k–n) The levels of the TNF‐α (k), IFN‐γ (l), IL‐10 (m), and IL‐4 (n) in tumor tissues after different treatments. Data are presented as mean ± SD (*n* = 3/group), ^*^ *p* < 0.05, ^**^ *p* < 0.01, and n.s. (not significant). o) H&E staining, ROS staining, TUNEL staining, and HMGB1 staining of tumor tissues in different groups. Scale bar, 200 µm. p) Western blot analysis of γ‐H_2_AX in the nucleus of tumor cells after different treatments. q) Schematic illustration of the anti‐tumor immune response triggered by PMIA + L mediated ROS generation and Pt^2+^ ions releasing.

We further assessed the impact of plasmonic catalysis‐driven TME modulation of PMIA for photometalloimmunotherapy in vivo (**Figure** **6**a). PMIA + L treatment strongly suppressed tumor growth compared to other experimental groups (Figure 6b,c). To gain insights into the immune response triggered by PMIA, we meticulously analyzed tumor‐infiltrating immune cells and related cytokines in serum following various treatments. As shown in Figure 6d,e, PMIA + L treatment obviously promoted DC (CD80^+^CD86^+^) maturation in tumor‐draining lymph nodes (TDLNs). Particularly, the remarkable up‐regulation of T cells (CD3^+^CD4^+^T and CD3^+^CD8^+^T cells) (Figure 6f,g) and M1‐type TAMs (stained with F4/80^+^CD86^+^) (Figure S14, Supporting Information), as well as the down‐regulation of Tregs (stained with CD3^+^CD4^+^Foxp3^+^) (Figure 6h), MDSC cells (stained with CD3^+^CD11b^+^CDGr‐1^+^) (Figure 6i) and M2‐type TAMs (Figure 6j) were observed in PMIA + L‐treated tumor tissues. Moreover, the serum levels of antitumor cytokines in terms of tumor necrosis factor α (TNF‐α) (Figure 6k) and interferon gamma (IFN‐γ) (Figure 6l) markedly rose, while anti‐inflammatory cytokines such as IL‐10 (Figure 6m) and IL‐4 (Figure 6n) decreased in PMIA + L‐treated groups. These results further indicate the effective reversal of tumor immunosuppression by PMIA + L treatment.^[^ ^36^ ^]^ Next, ROS staining, terminal deoxynucleotidyl transferase‐mediated dUTP nick end‐labeling (TUNEL), and HMGB1 staining results showed that, upon NIR laser irradiation, PMIA induced severe ROS generation, substantial cell apoptosis and ICD (Figure 6o).^[^ ^37^ ^]^ We further confirmed PMIA + L induce more severe DNA damage in tumor tissue via western blot analysis (Figure 6p). Additionally, no obvious change in body weights (Figure S15, Supporting Information), biochemical analysis (Figure S16, Supporting Information), blood tests (Figure S17, Supporting Information), and histopathological analysis (Figure S18, Supporting Information) in mice treated with PMIA underscored its excellent biosafety. These results demonstrate the efficient plasmonic catalysis‐driven antitumor activity of PMIA under NIR laser irradiation, achieved through simultaneous ROS‐induced ICD induction and Pt^2+^ ion release‐mediated ICD enhancement for robust cancer photometalloimmunotherapy (Figure 6q).

**Figure 7:**
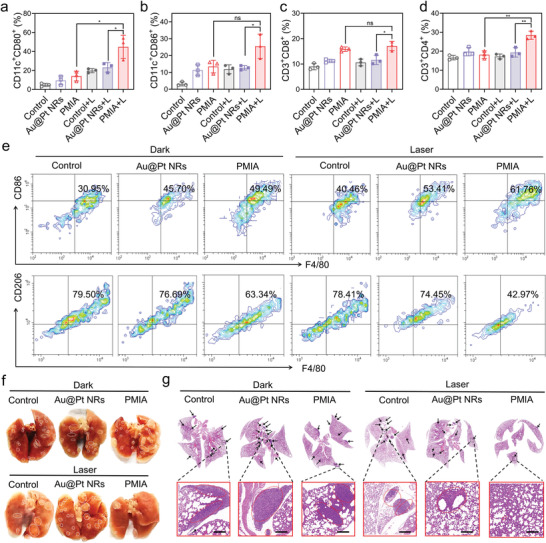
Evaluation of the antitumor efficacy of anisotropic plasmonic PMIA on distant tumor and lung metastasis. a,b) Analysis of DC maturation in tumor‐draining lymph nodes after different treatments by flow cytometry. Data are presented as mean ± SD (*n* = 3/group), ^*^ *p* < 0.05, ^**^ *p* < 0.01, and n.s. (not significant). c,d) Proportion of CD8^+^T cells (c) and CD4^+^T cells (d) in distant tumors after different treatments detected by flow cytometry. Data are presented as mean ± SD (*n* = 3/group), ^*^ *p* < 0.05, and n.s. (not significant). e) The levels of M2 macrophages (F4/80^+^CD206^+^) and M1 macrophages (F4/80^+^CD80^+^) in distant tumor under different treatments detected by flow cytometry. f,g) The photographs (f) and H&E staining images of lung (g). The tumor metastatic nodules are indicated by the arrows. Scale bar, 250 µm. The tumor metastatic nodules are indicated by the dashed circles.

We investigated the anti‐tumor effects of PMIA in combination with NIR laser irradiation on distant tumor growth and lung metastasis in mouse models. As demonstrated in **Figure** **7**a,b, PMIA + L treatment significantly enhanced the DC maturation, which is conductive to the initiation of T‐cell‐mediated immune response.^[^ ^38^ ^]^ Further, CD8^+^T and CD4^+^T cells in the distant tumors were analyzed. Flow cytometry analysis displayed that proportions of CD8^+^T and CD4^+^T cells were remarkedly enhanced in the distant tumor after PMIA + L treatment (Figure 7c,d), suggesting the significant antitumor immune response induced by PMIA under NIR laser irradiation. Then, the phenotypic change of TAMs in distant tumors was carefully examined. After different treatments, the levels of M2‐phenotype macrophages (F4/80^+^CD206^+^) and M1‐phenotype macrophages (F4/80^+^CD80^+^) in PMIA + L group showed a clear decrease and increase, respectively (Figure 7e). In addition, there was almost no tumor metastasis found in the lungs of mice in PMIA + L group, indicating the effective tumor metastasis inhibition (Figure 7f,g). These results clearly show that PMIA, when combined with NIR irradiation, efficiently inhibits distant tumor growth and lung metastasis.

## 3 Conclusion

In summary, to overcome the challenges associated with NIR light‐triggered photoimmunotherapy, we meticulously engineered an anisotropic dumbbell‐shaped photo‐/metallo dual‐mode immunotherapeutic agent (PMIA). Through precise control of the heteroepitaxial multi‐site growth of starry Pt nanoclusters on Au NRs, we established a potent framework for cancer photometalloimmunotherapy. Our experimental findings, corroborated by DFT and FDTD calculations, validated that the engineered Pt nanoclusters, arranged in this distinctive configuration, not only facilitated radial electron distribution polarization and enhanced the local electric field but also provided an abundance of active reaction sites. This conducive environment facilitated the generation of abundant ROS and the release of Pt^2+^ ions. Leveraging these characteristics, PMIA demonstrated remarkable efficacy in inducing intranuclear DNA damage and synergistically amplifying ICD, thereby activating systemic immune responses. Notably, this effect was evident even under low‐power NIR irradiation (0.05 W cm^−2^), showcasing its robust performance in treating both primary and distant tumors while effectively inhibiting tumor metastasis in vivo. This study represents a significant advancement in our understanding of photo‐/metallo dual‐mode immunotherapeutic agents, offering valuable insights that can guide the development of highly efficient photometalloimmunotherapy for clinical translation.

## 4 Experimental Section

### Preparation of Au Nanorods (NRs)

Au NRs were synthesized by a seed‐mediated method.^[^ ^17^ ^]^ Briefly, the seed solution was made by adding a freshly prepared aqueous solution of NaBH_4_ (50 µL, 0.01 m) into a mixture solution composed of HAuCl_4_·3H_2_O (50 µL, 0.01 m) and CTAB (1 mL, 0.2 m). The solution was stirred vigorously for 2 min and aged at room temperature for 1.5 h before use. In 100 mL of deionized water (Millipore, 60 °C), 3.6 g of CTAB and 0.44 g of 5‐bromosalicylic acid were dissolved to make the growth solution. Then 1.92 mL of 0.01 m AgNO_3_ was added. After 15 min, 10 mL of 0.01 m HAuCl_4_·3H_2_O solution was added. After gentle stirring for 15 min, 0.512 mL of 0.1 m ascorbic acid (AA) was added for 30 s until the mixture became colorless. 1 mL seed solution was added to the entire growth solution. The mixture was stirred for 2 min and left undisturbed at 27 °C for 12 h. The color of the growth solution slowly changed from colorless to wine, indicating the growth of Au NRs. The as‐made Au NRs were used for subsequent multi‐site Pt growth without purification treatment.

### Tuning the Multi‐Site Pt Growth on Au NRs

For the synthesis of AuPt HSs,^[^ ^18^ ^]^ 0.036 g of CTAB and 0.004 g of 5‐bromosalicylic acid in 2 mL of deionized water, H_2_PtCl_6_ (0.002 m), 50 µL of AA (0.1 m), and 32 µL of HCl (0.01 m) were added into 2 mL of as‐made Au NRs. After stirring for 2 min, the mixture was left undisturbed for 12 h at 30 °C. To fine‐tuning the multi‐site Pt growth on Au NRs, amount of H_2_PtCl_6_ (0.002 m; 100 µL, 200 µL, 300 µL and 400 µL) was adjusted to construct a series of AuPt HSs (from AuPt‐1 HSs to AuPt‐4 HSs, respectively).

### Preparation of Au@Pt NRs

Briefly, the solution that was prepared by dissolving CTAB (0.036 g) and 5‐bromosalicylic acid (0.004 g) in 2 mL of deionized water was added into 2 mL of the as‐made Au‐NRs. Then, 600 µL of H_2_PtCl_6_ (0.002 m) and subsequently 32 µL of HCl (0.01 m) were added to the mixture. After stirring for 2 min, the mixture was left undisturbed for 12 h at 30 °C.

### Surface Modification of AuPt NRs

Briefly, 60 mg of mPEG_5k_‐SH was dissolved in 3 mL of deionized water, followed by adding 5 mL of AuPt HSs. The reaction aqueous solution was stirred for 24 h. The excess mPEG_5k_‐SH was removed by centrifugation. The obtained AuPt HSs‐PEG were then resuspended in deionized water for further use.^[^ ^39^ ^]^

### Animal Ethics Statement

All animal experiment protocols were approved by the Medical Ethics Committee of Shanxi Medical University (2019SLL188) and Animal Ethical Committee of Zhejiang University (approval number: 19 393). All animal studies were performed in compliance with relevant ethical regulations.

### Statistical Analysis

All data were presented as the mean ± SD from a minimum of three independent experiments. All data were statistically analyzed by using GraphPad Prism Software Version 8.0.2 (GraphPad Prism, USA), Origin 2018 (OriginLab Corporation, USA) and/or Image J (version 1.52a). Each experiment was performed at least three times (n≥3), and the specific sample size (n) was clarified in the legends of figures. For statistical comparison, t‐test was performed to compare data between two groups. These results were considered significant at ^*^ *p* < 0.05, ^**^ *p* < 0.01, ^***^ *p* < 0.001, ^****^ *p* < 0.0001 and n.s. (not significant).

## Conflict of Interest

The authors declare no conflict of interest.

## Supporting information

Supporting Information

## Data Availability

The data that support the findings of this study are available from the corresponding author upon reasonable request.
