# Supplementary material for: Catalytic Dual‐Mode Immunotherapy: Anisotropic AuPt Heterostructure Decorated with Starry Pt Nanoclusters for Robust Cancer Photometalloimmunotherapy
Source: Adv Sci (Weinh). 2024 May 30;11(28):2403116. doi: 10.1002/advs.202403116 (PMC11267392; doi:10.1002/advs.202403116)
Supplement: Supplementary file 1 — Supporting Information [file ADVS-11-2403116-s001.docx]

Supporting Information

Catalytic Dual-Mode Immunotherapy: Anisotropic AuPt Heterostructure Decorated with Starry Pt Nanoclusters for Robust Cancer Photometalloimmunotherapy

Wei Bian, Xi Hu*, Ruixue Xiao, Rui Yao, Bo Zhang, Mingjian Zhu, Tianqi Liu, Yamin Liu, Jing Li, Peihua Lin, An Xie, Fangyuan Li*, and Daishun Ling*

Prof. W. Bian, Dr. J. Li

Department of Breast Surgery, First Hospital of Shanxi Medical University, Taiyuan, 030001, China

Prof. W. Bian, R. Yao, T. Liu

Key Laboratory of Cellular Physiology at Shanxi Medical University, Ministry of Education，Taiyuan 030000, China

R. Xiao, Dr. B. Zhang, Dr. Y. Liu, Dr. P. Lin, Prof. D. Ling

Frontiers Science Center for Transformative Molecules, School of Chemistry and Chemical Engineering, School of Biomedical Engineering, National Center for Translational Medicine, Shanghai Jiao Tong University, Shanghai 200240, China

E-mail: dsling@sjtu.edu.cn (D. Ling)

Prof. F. Li

Songjiang Institute and Songjiang Hospital, Shanghai Key Laboratory of Emotions and Affective Disorders, Shanghai Jiao Tong University School of Medicine, Shanghai 200025, China

Key Laboratory of Precision Diagnosis and Treatment for Hepatobiliary and Pancreatic Tumor of Zhejiang Province, Hangzhou 310009, China

E-mail: lfy@shsmu.edu.cn (F. Li)

Prof. X. Hu, A. Xie

School of Pharmacy, Anhui University of Chinese Medicine, Hefei 230012, China

E-mail: huxi@ahtcm.edu.cn (X. Hu)

M. Zhu

Institute of Pharmaceutics, Hangzhou Institute of Innovative Medicine, College of Pharmaceutical Sciences, Zhejiang University, Hangzhou 310058, China

Dr. B. Zhang, Prof. D. Ling

WLA Laboratories, Shanghai 201203, China

Keywords: dumbbell-like heterostructures, platinum nanoclusters, cancer photometalloimmunotherapy, immunogenic cell death, ion release

**Supplementary Materials**

*Chemicals:* Gold chloride trihydrate (HAuCl_4_·3H_2_O), sodium borohydride (NaBH_4_), ascorbic acid (AA), chloroplatinic acid (H_2_PtCl_6_), 5-bromosalicylic acid (5-BrSA), 3,3’,5,5’-tetramethylbenzidine (TMB), 1,3-diphenylisobenzofuran (DPBF), carotene, mannite, 5,5-dimethyl-1-pyrroline-N-oxide (DMPO), 2,2,6,6-tetramethylpiperidine (TEMP), sodium acetate (NaAc), acetate, rhodamine B isothiocyanate (RITC), and 3-(4,5-Dimethylthiazol-2-yl)-2,5-diphenyltetrazolium bromide (MTT) were purchased from Aladdin Industrial Inc. (Shanghai, China). N-acetyl cysteine (NAC) was purchased from Sigma-Aldrich. 5-tertbutoxycarbonyl-5methyl-1-pyrroline N-oxide (BMPO) was bought from APExBIO (Houston, USA). Superoxide dismutase (SOD) was purchased from Shanghai Yuanye Bio-Technology Co., Ltd. (Shanghai, China). Cetyltrimethylammonium bromide (CTAB), silver nitrate (AgNO_3_), hydrogen peroxide (H_2_O_2_), and dimethyl sulfoxide (DMSO) were purchased from Sinopharm Chemical Reagent Co., Ltd. (Shanghai, China). Methoxy poly (ethylene glycol)-5000-thiol (mPEG_5K_-SH) and amine poly (ethylene glycol)-5000 thiol (NH_2_-PEG_5K_-SH) were purchased from Punsore Biotechnology Company (China). Fetal bovine serum (FBS) was purchased from Sijiqing Biologic Co., Ltd. (Hangzhou, China). RPMI1640 was purchased from Cienry Biotechnology Co., Ltd. (Huzhou, China). ATP assay kit, JC-1 mitochondrial membrane potential assay kit, 2’,7’-dichlorofluorescein diacetate (DCFH-DA) were purchased from Beyotime Institute of Biotechnology (Haimen, China). Annexin VFITC/PI apoptosis detection kit was obtained from Nanjing Jiancheng Bioengineering Institute (Nanjing, China). HMGB1 antibody and CRT antibody were purchased from Proteintech (Wuhan, China). Anti-mouse F4/80 antibody, anti-mouse CD80 antibody, anti-mouse CD86 antibody, anti-mouse CD206 antibody, anti-mouse CD3 antibody, anti-mouse CD8 antibody, anti-mouse CD11c antibody, anti-mouse CD11b antibody, anti-mouse CD25 antibody were purchased from BioLegend (San Diego, USA). The anti-mouse γ-H_2_AX antibody, TNF-α Elisa kit, INF-γ Elisa kit, IL-4 Elisa kit, and IL-10 Elisa kit were purchased from Boster (Wuhan, China). All reagents and solvents were obtained commercially and used without further purification.

*Characterizations:* Transmission electron microscopy (TEM) images were acquired on a transmission electron microscope (Hitachi HT7700, Japan). UV-vis absorption spectra were obtained via a Shimadzu UV-2450 spectrophotometer. X-ray diffraction (XRD) patterns were obtained using an X-ray diffractometer (PANalytical B.V. X-pert Powder, Netherlands). High-angle annular dark-field scanning transmission electron microscopy (HAADF-STEM) images were obtained via using a spherical aberration-corrected Titan ChemiSTEM microscope. The mass ratios of Au and Pt in the products were determined by an inductive coupling plasma mass spectrometer (ICP-MS) system. The X-ray photoelectron spectra (XPS) were conducted on a Thermo Scientific ESCALAB 250 Xi XPS system. The hydrodynamic size and zeta potential were measured by using the Zetasizer Nano ZS90 (Malvern Instruments, UK). Electron spin resonance (ESR) spectra were measured by the Bruker A300 EPR spectrometer.

*Electrochemical analysis*: Electrochemical experiments were performed on a standard three-electrode cell connected with an electrochemical workstation (CH Instruments, Inc, Shanghai) at RT. Ag/AgCl (4 M KCl) and Pt sheet were used as the reference electrode and counter electrode, respectively.^[1]^ The working electrode was modified with different AuPt-1 HSs, AuPt-2 HSs, AuPt-3 HSs, AuPt-4 HSs, and Au@Pt NRs for electrochemical analysis. For preparing the working electrode, Nafion (1.5 μL) and isopropanol (10 μL) were added to the AuPt HSs (88.5 μL) and then sonicated for 30 min. Subsequently, the mixture was poured onto the freshly polished glassy carbon electrode (0.07065 cm^2^) and dried. The modified working electrodes were scanned in an N_2_-purged H_2_SO_4_ solution (0.5 M) with or without NIR irradiation (808 nm, 0.5 W cm^−2^).

*Catalytic activities evaluation:* TMB was employed to evaluate the catalytic activities of AuPt HSs with/without 808 nm laser irradiation. In detail, AuPt HSs was added to a HAc/NaAc buffer solution (0.2 M, pH = 5.0) containing H_2_O_2_ (10 mM) with a total volume of 2 mL. To exclude the photo-absorption difference of AuPt HSs, their concentrations were modulated to make absorbance values at 808 nm the same, i.e., the Au concentrations were 0.31 mg mL^−1^ (AuPt-1 HSs), 0.18 mg mL^−1^ (AuPt-2 HSs), 0.16 mg mL^−1^ (AuPt-3 HSs) and 0.19 mg mL^−1^ (AuPt-4 HSs), respectively. Then, 20 μL of TMB (50 mM) was added to the above mixture to initiate the oxidizing reaction of TMB with/without NIR irradiation (808 nm, 0.5 W cm^−2^). The oxidation process of TMB was recorded at different time points by UV-vis detection at 652 nm.^[2]^

2.7 mg of DPBF was dissolved in the 1 mL of ethanol, 20 μL of DPBF ethanol solution, 200 μL of H_2_O_2_ (100 mM), AuPt-3 HSs (i.e., PMIA; 50 μg mL^−1^ of Au), and 100 μL of scavenger were dispersed in the mixed solution (V_ethanol_:V_water_ = 6:4) with a total volume of 2 mL. The scavengers were involved in superoxide dismutase (4,000 U mL^-1^, a ^•^O_2_^−^ scavenger), carotene (2 mg mL^-1^, a ^1^O_2_ scavenger), and mannitol (50 mM, a •OH scavenger).^[3]^ The absorbance value of DPBF at 410 nm was detected under NIR irradiation (808 nm, 0.5 W cm^−2^) by a UV-vis spectroscopy.

*ESR measurements*: ESR measurements were carried out for the detection of •OH, ^1^O_2_, and •O_2_^−^ with 808 nm laser irradiation (0.5 W cm^−2^). For ^1^O_2_ measurement. 10 μL of Au NRs, Au@Pt NRs, or PMIA (50 μg mL^−1^ of Au) was added to the mixture of TEMP (1 M, 57  μL) in DMSO and HAc/NaAc solution (0.2 M, 143 μL, pH = 5.0). The solution were mixed and exposed to 808 nm laser irradiation (0.5 W cm^−2^) for 5 min before ESR measurement. For •O_2_^−^ measurement. 10 μL of Au NRs, Au@Pt NRs, and PMIA (50 μg mL^−1^ of Au) was added to the mixture of DMPO (0.2 M, 100  μL) in DMSO and HAc/NaAc solution (0.2 M, 100 μL, pH = 5.0). The solution was mixed and exposed to 808 nm laser irradiation (0.5 W cm^−2^) for 5 min before ESR measurement. For •OH measurement, 10 μL of Au NRs, Au@Pt NRs, and PMIA (50 μg mL^−1^ of Au) was added to the mixture of BMPO (0.2 M, 100 μL) and HAc/NaAc solution (0.2 M, 100 μL, pH = 5.0) containing 10  mM H_2_O_2_. The solution was mixed and exposed to 808 nm laser irradiation (0.5 W cm^−2^) for 5 min before ESR measurement.

*Pt^2+^ ions release:* 1 mL of PBS (pH 5.5, 10 mM) containing 200 μg of AuPt HSs was placed in a dialysis bag with a cut-off of 3500 Da and dialyzed against 10 mL of PBS upon 808 nm irradiation (0.5 W cm^−2^, 5 min) or in the dark condition. The concentration of the released Pt^2+^ ions at 37 °C after 24 h was quantified by using the ICP-MS. Furthermore, the accumulated Pt^2+^ ions release from PMIA in PBS (pH 7.4, 10 mM), PBS (pH 6.5, 10 mM), PBS (pH 5.5, 10 mM), and H_2_O_2_/PBS (pH 5.5, 10 mM) with 808 nm laser irradiation (0.5 W cm^−2^, 5 min) were also determined using the above-mentioned method.

*DFT calculation:* Au and Pt bulk structure were obtained from Materials Project (https://legacy.materialsproject.org/, Materials ID: mp-81 and mp-126) and optimized. Then the Au bulk structure was cleaved to the (200) surface, the Pt bulk structure was cleaved to the (111) surface. The Au bulk structure was modeled with the (3×3) periodically repeated supercell, consisting of 2 atomic layers, with a vacuum space of 15 Å. The heterojunction structures of Pt on Au surfaces were constructed using Vaspkit package and relaxed. The calculations were performed using the DFT method in combination with Standard solid-state pseudopotentials (SSSP) Efficiency potentials and Perdew-Burke-Ernzerhof (PBE) exchange-correlation functional as implemented in Quantum Espresso package. The kinetic energy cutoff for the plane-wave-basis set was set as 45 Ry. For all geometry optimizations, the energy convergence criterion for the electronic self-consistent loop and the ionic relaxation loop were set as 1.0×10^−5^ and 1.0×10^−4^, respectively. 2×2×1 Monkhorst-Pack k-point mesh samplings were used during the DFT calculation process.

*FDTD simulation:* The electric field distribution was calculated using MEEP, a free and open-source software package for electromagnetics simulation via the finite-difference time-domain (FDTD) method spanning a broad range of applications.^[4]^ The optical constants of Au were adopted from tabulated value for bulk gold measured by Johnson and Christy.^[5]^ The optical constants of Pt and H_2_O were adopted from tabulated value measured by Palik.^[6]^ The size of the nanorod was taken to match the average value. Specifically, Au NR was modeled as a cylinder capped two half-spheres at the ends. For the model of dumbbell-like AuPt heterostructure, Pt spheres were located at both ends of the Au NR. For the model of the core-shell AuPt heterostructure, a thin layer Pt was homogeneously located on the surface of the Au NR. For the model of anisotropic dumbbell-shaped PMIA, on the basis of the dumbbell-like AuPt heterostructure, multiple tiny Pt spheres were set onto the AuPt HSs side surface.

*Preparation of RITC-labeled mPEG_5K_-SH:* 10 mg of RITC was dissolved in 0.5 mL of ethanol and then added to NH_2_-mPEG_5K_-SH solution in distilled water (100 mg mL^−1^, 2 mL). After stirring at room temperature (RT) for 12 h, the filtered solution was dialyzed in a dialysis bag with a cut-off of 3500 Da against distilled water for 48 h to obtain RITC-labeled mPEG_5K_-SH.^[1]^

*Preparation of RITC-labeled PMIA:* 0.2 mg of PMIA and 10 mg of RITC-labeled mPEG_5K_-SH were mixed with 2 mL of distilled water and stirred at RT for 24 h. The RITC-labeled PMIA were centrifugated and rinsed three times with distilled water for further use.

*Cell culture:* 4T1 murine breast cancer cells and RAW 264.7 macrophages were obtained from China Infrastructure of Cell Line Resources (Beijing, China). The cells were cultured in RPMI1640 medium containing 10% FBS and 100 U mL^−1^ mixed penicillin/streptomycin at 37°C under a humidified atmosphere with 5% CO_2_. The cell culture medium was replaced with fresh one every other day unless otherwise noted.

*Cellular uptake*: 4T1 cells were seeded in a 12-well plate at 1 × 10^5^ cells and incubated for 12 hours. Then, the cells were treated with 20 μL of RITC-labeled PMIA (1 mg mL^-1^, equivalent to 20 μg mL^−1^ of Au) for CLSM detection. Besides, after incubation for 1 h, 2 h, 4 h, 6 h, 8 h, 12 h, and 24 h, the cells were washed with PBS three times, digested, and counted, followed by thorough dissolution with aqua regia. Subsequently, after dilution and filtration, the Au concentrations were quantified by ICP-MS analysis.

*Cell viability assays in vitro*: 4T1 cells were seeded in 96-well plates at 1 × 10^4^ cells/well and incubated for 12 h. Cells were then co-incubated with AuPt HSs or Au@Pt NRs in the presence or absence of 808 nm laser irradiation (0.5 W cm^−2^) for 5 min. Then cells were treated with MTT solutions (0.5 mg mL^−1^). After incubation for 4 h, the media were replaced with DMSO and the absorbance of each well was measured at 570 nm via a microplate reader (Bio-Rad, Hercules, California, USA). Moreover, viability of 4T1 cell after the treatment of PMIA upon NIR irradiation (0.05 Wcm^−2^) for 10 min was also evaluated.

*Intracellular ROS detection*: 4T1 cells were seeded in confocal plates at 1 × 10^5^ cells/plate, and treated with PBS, PMIA and Au@Pt NRs (25 μg mL^−1^ of Au) for 12 h in the presence or absence of 808 nm laser irradiation (0.05 W cm^−2^, 10 min), respectively. Then, the cells were then incubated with 500 μL RPMI1640 containing 1 μM DCFH-DA for 20 min and subsequently washed with PBS (pH 7.4) for further CLSM detection.

*JC-1 staining*: 4T1 cells were seeded in confocal plates (1 × 10^5^ cells/plate), and treated with PBS, PMIA, and Au@Pt NRs (25 μg mL^−1^ of Au) in the presence or absence of 808 nm laser irradiation (0.05 W cm^−2^, 10 min), respectively. Then, the cells were stained with JC-1 working solution for 20 min and washed with PBS for further CLSM detection.

*Flow cytometry analysis*: 4T1 cells were seeded in 6-well plates (2 × 10^5^ cells/plate), and treated with PBS, PMIA or Au@Pt NRs (25 μg mL^−1^ of Au) in the presence or absence of 808 nm laser irradiation (0.05 W cm^−2^, 10 min), respectively. After incubation for 24 h, cells were suspended and stained with propidium iodide and FITC-Annexin V reagent for flow cytometry analysis (Becton Dickinson, USA).

*Immunofluorescence detection of HMGB1 and CRT in vitro*: 4T1 cells were seeded in confocal plates (1 × 10^5^ cells/plate) and treated with PBS, PMIA, or Au@Pt NRs (25 μg mL^−1^ of Au), respectively, in the presence or absence of 808 nm laser irradiation (0.05 W cm^−2^, 10 min). The cells were then fixed with 4% paraformaldehyde, blocked, and incubated with HMGB1 mouse monoclonal antibody or CRT mouse monoclonal antibody, respectively. Then, the cells were incubated with FITC-labeled goat anti-mouse IgG (H + L), and stained with DAPI for further CLSM analysis.

*In vitro dendritic cell stimulation:* For in vitro DC stimulation experiments, PMIA or Au@Pt NRs at a dose of 25 µg mL^−1^ were incubated with 4T1 cells in the presence or absence of 808 nm laser irradiation, residues of 4T1 cells after different treatments were introduced into DC culture using a transwell system. After various treatments, DCs were stained with anti-CD86-PE, anti-CD80-APC antibody and analyzed by a flow cytometry.

*Anti-tumor effect in vivo*: For the unilateral tumor model, 4T1 cells (1 × 10^6^ cells) were injected into the 5-week female BALB/c mouse to establish the breast cancer mice models. After 7 days, the mice were randomly divided into six groups (n = 5) for different treatments, including PBS, PMIA, Au@Pt NRs, PBS + Laser (L), PMIA + L and Au@Pt NRs + L. PBS, PMIA, or Au@Pt NRs (10 mg kg^−1^) were intratumorally injected into the tumor-bearing mice in each group. The 808 nm laser (50 mW cm^−2^, 10 min) was applied to trigger the photocatalytic therapy of the tumor-bearing mice. The tumor volumes and the body weights of the mice were recorded every other day. For the bilateral tumor model, 4T1 cells (1 × 10^6^ cells) were injected into the left flank of the 5-week female BALB/c mouse to establish the primary tumor. For the second tumor establishment, 4T1 cells (4 × 10^5^ cells) were injected into the right flank of each mouse 5 days later. Then, the mice were randomly divided into six groups (n = 5) for different treatments, including PBS, PMIA, Au@Pt NRs, PBS + L, PMIA + L and Au@Pt NRs + L. PBS, PMIA, or Au@Pt NRs (10 mg kg^-1^) were intratumorally injected into the tumor-bearing mice in each group. The laser treatment (50 mW cm^−2^, 10 min) was applied to the primary tumor to trigger ICD. Afterwards, the body weights and tumor volumes in both flanks of mice were recorded every other day.

*Immune cells analysis*: 4T1 tumors or tumor draining lymph nodes (tdLNs) were collected and digested in RPMI1640 media that contained collagenase and DNase at 37°C for 1 h. Furthermore, for DCs stimulation analysis, DCs harvested from tdLNs were stained with FITC-CD11c, APC-CD86, and PE-CD80 for flow cytometry assay. And cells in tumors were stained with PE-CD3, FITC-CD4, and APC-CD8 to investigate the CTLs levels via flow cytometry assay.

*Biosafety test in vivo:* Healthy mice were intravenously injected with PMIA (10 mg kg^−1^). PBS-treated mice were set as control groups. The body weights of mice were recorded every other day. On day 21 after administration, mice were sacrificed to collect blood, and the main organs (heart, liver, spleen, lung and kidneys) of mice were also collected. The blood and main organs were used for further tests and histological staining, respectively.

**Supplementary Figures**


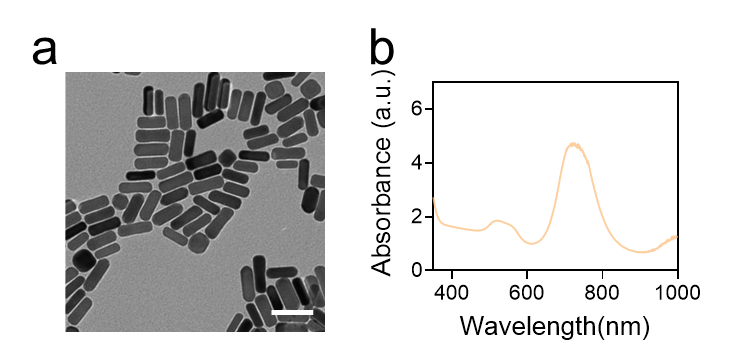


**Figure S1.** (a) The TEM image of Au nanorods (NRs). Scale bar is 50 nm. (b) UV-vis absorption spectra of Au NRs.


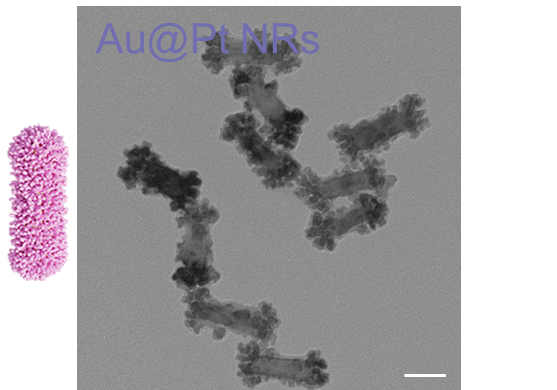


**Figure S2.** The TEM image of Au@Pt NRs. Scale bar is 25 nm.

**
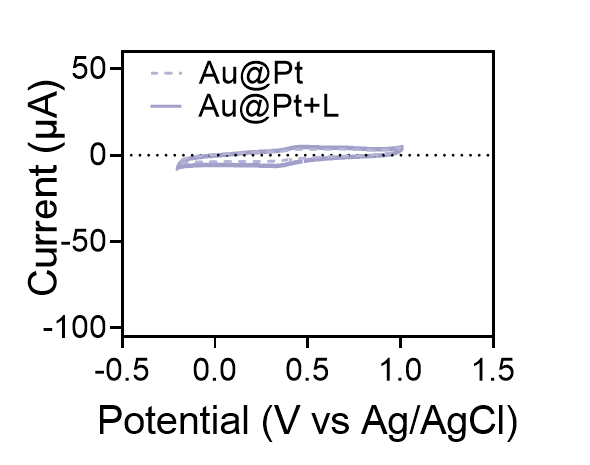
**

**Figure S3.** CV curves of Au@Pt NRs with (solid purple line) or without (dotted purple line) 808 nm laser irradiation.

**Figure S4.** CV curves of Au NRs with (red line) or without (black line) 808 nm laser irradiation.

**Figure S5.** UV-vis NIR absorption spectra of a series of AuPt HSs samples.


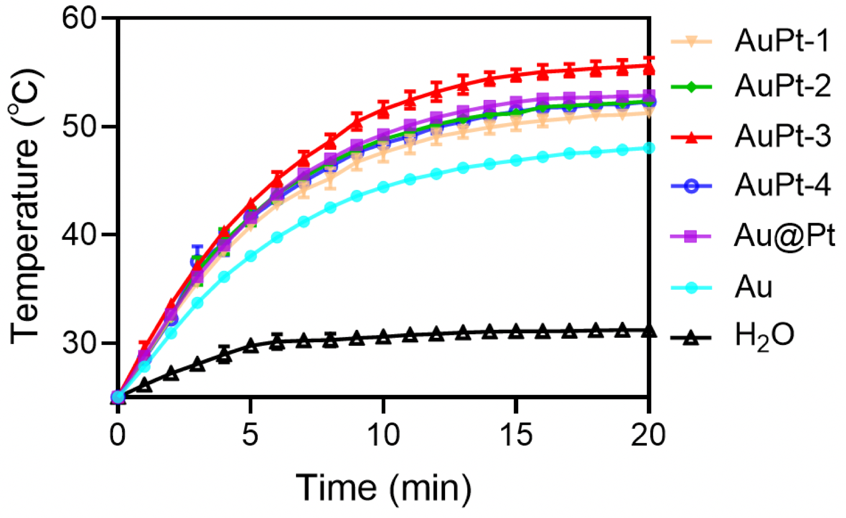


**Figure S6.** Photothermal curves of AuPt HSs, Au NRs, and Au@Pt NRs in aqueous solution irradiated by 808 nm laser (0.5 W cm^-2^, 20 min). Data are presented as mean ± SD (n = 3/group).


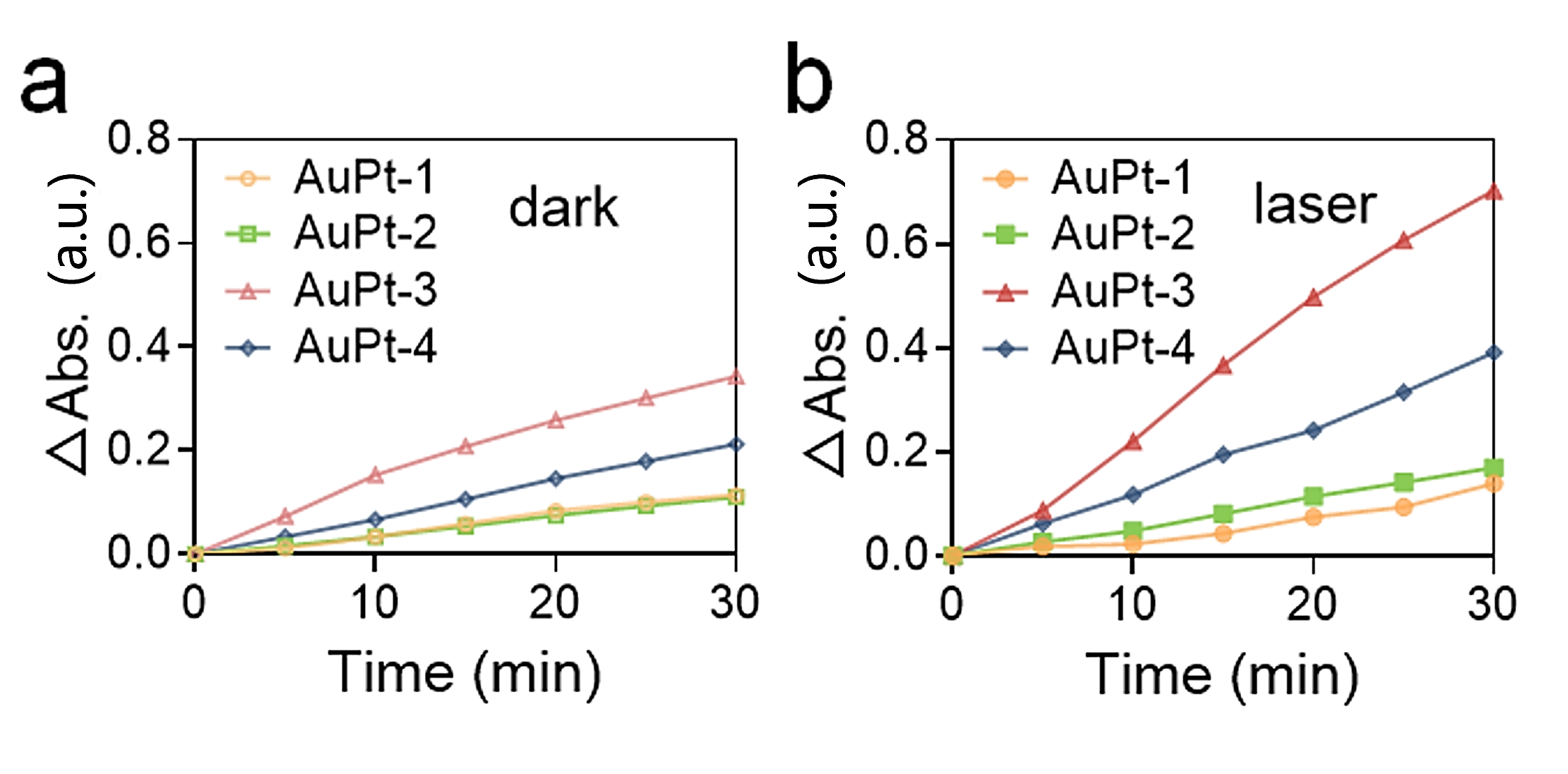


**Figure S7.** Time-course absorbance of TMB oxidation product at 652 nm in the presence of AuPt HSs and H_2_O_2_ without (a) and with (b) 808 nm laser irradiation.


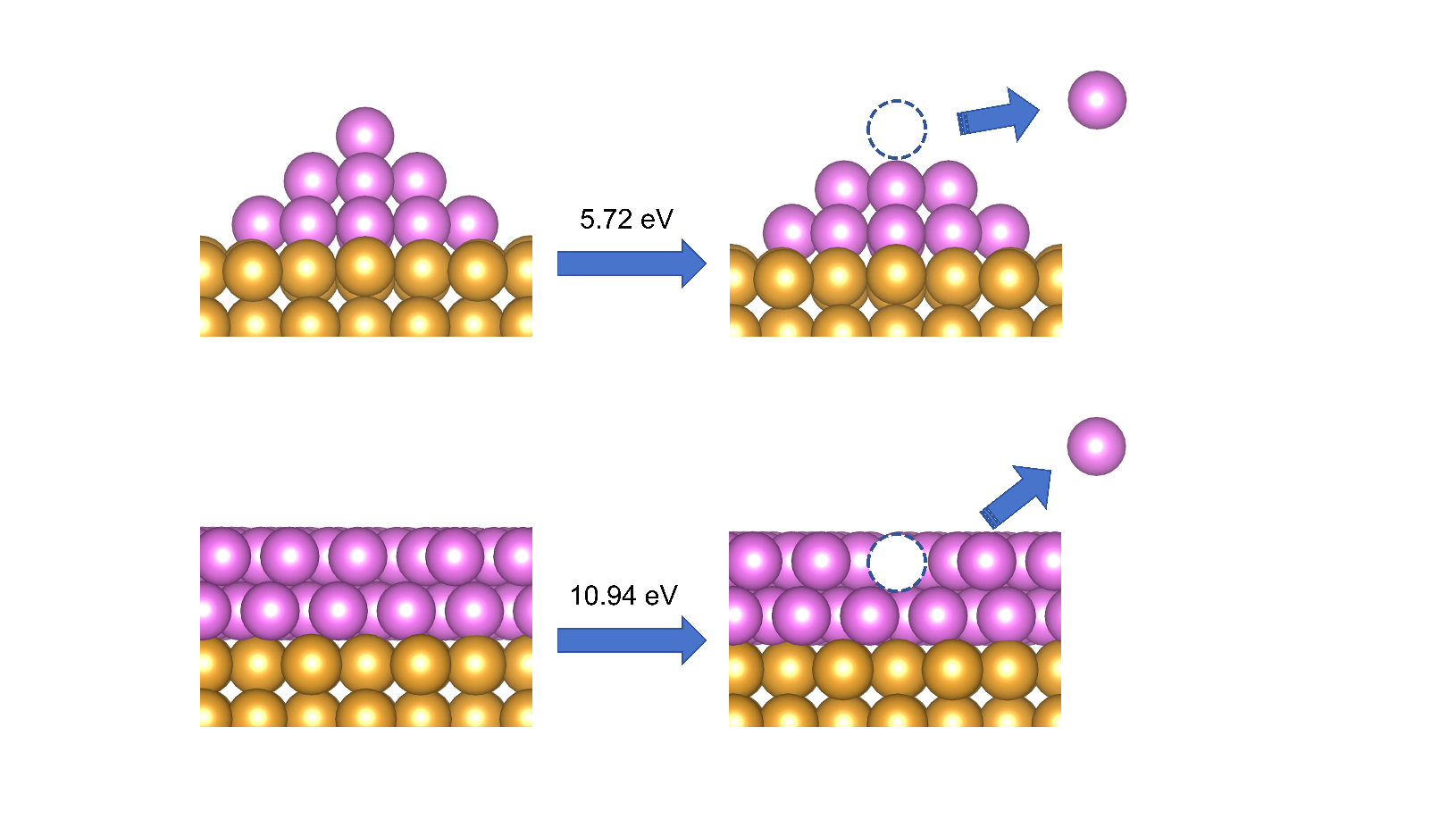


**Figure S8.** The schematic diagram and desorption energy of Pt ions on AuPt-3 HSs and Au@Pt NRs calculated by DFT method.


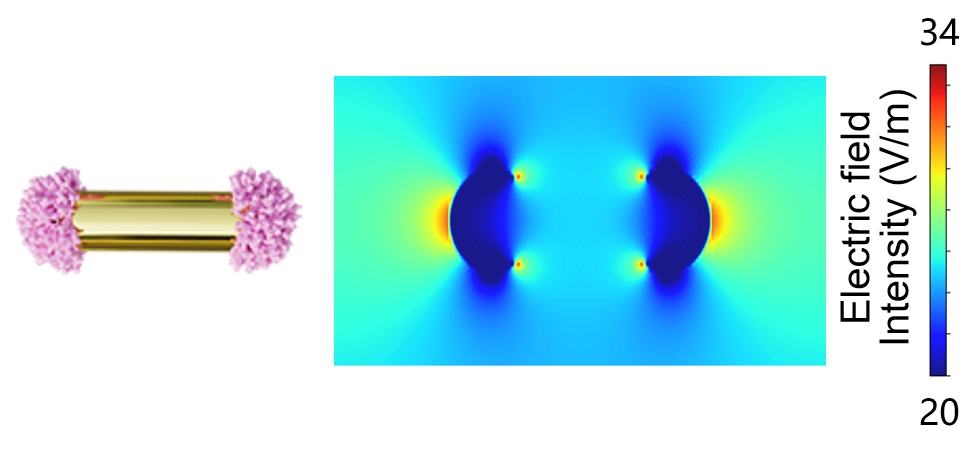


**Figure S9.** FDTD analysis of AuPt-2 HSs, lower electric field intensity only on the ends.


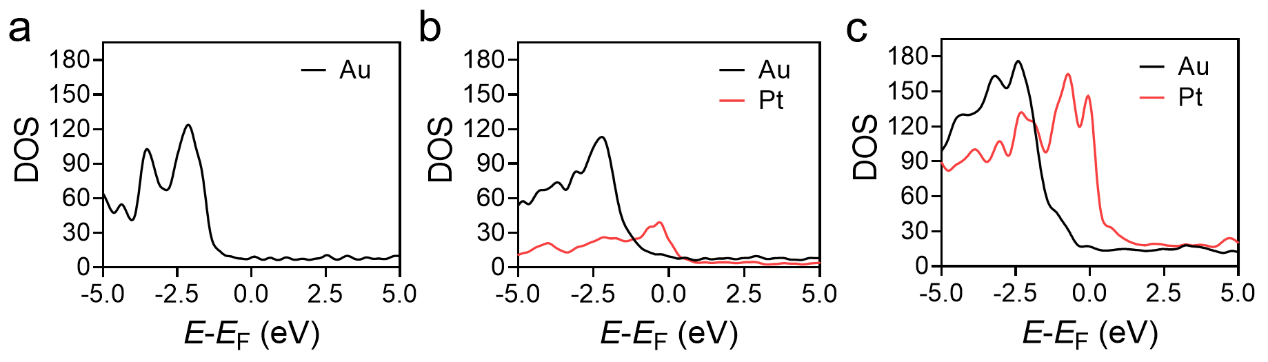


**Figure S10.** Density of states (DOS) analysis of all atoms on Au NR (a), PMIA (b) and Au@Pt core-shell NRs (c), respectively.


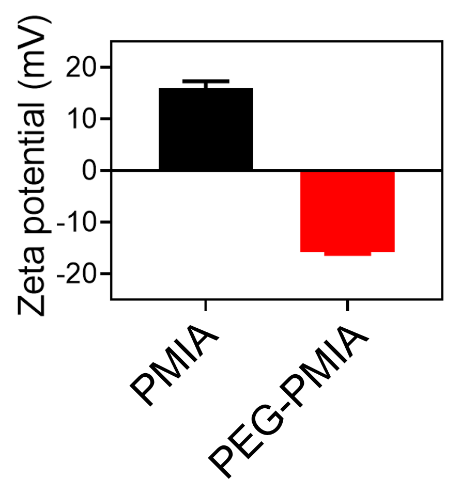


**Figure S11.** Zeta potentials of as-synthesized PMIA (CTAB-PMIA) and PEG-PMIA. The surface charge of PMIA switched from 15.93 mV to -15.76 mV after PEGylation. Data are presented as mean ± SD (n =  3/group).

**Figure S12.** Viabilities of 4T1 cells after incubation with Au@Pt NRs without and with 808 nm laser irradiation (0.5 W cm^−2^, 5 min). Data are presented as mean ± SD (n =  5/group).


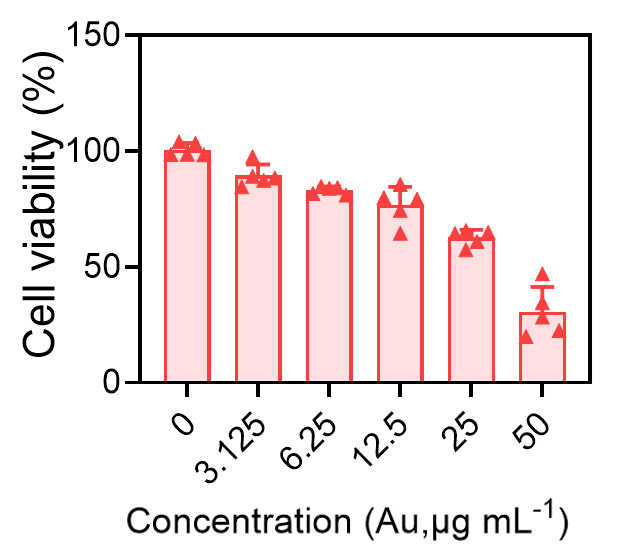


**Figure S13.** Viabilities of 4T1 cells after incubation with PMIA with 808 nm laser irradiation (0.5 W cm^−2^, 5 min) in an ice condition. Data are presented as mean ± SD (n =  5/group).


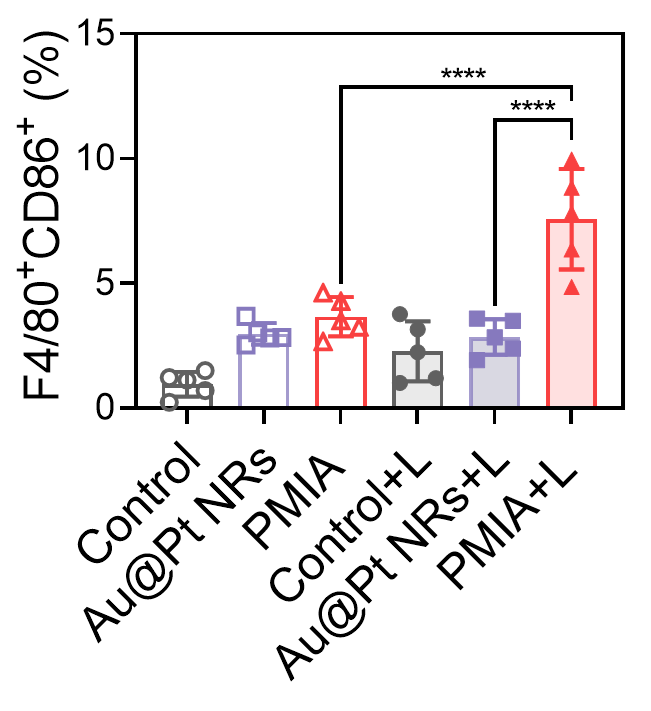


**Figure S14.** Proportion of M1-TAM (stained with F4/80^+^CD86^+^) after different treatments detected by a flow cytometry. Data are presented as mean ± SD (n =  5/group), ^****^p < 0.0001.


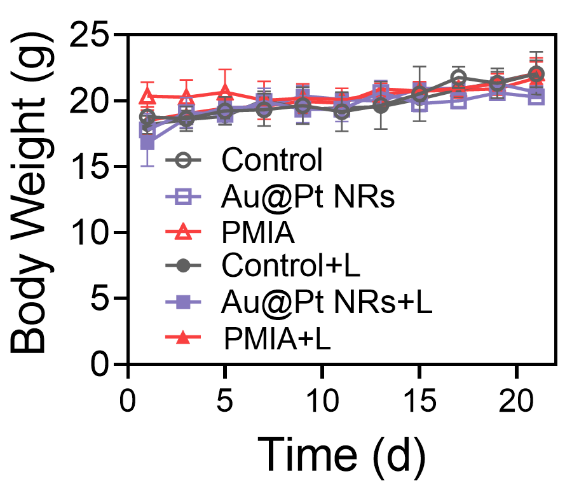


**Figure S15.** Body weights of 4T1 tumor-bearing mice with different treatments. Data are presented as mean ± SD (n = 3/group).


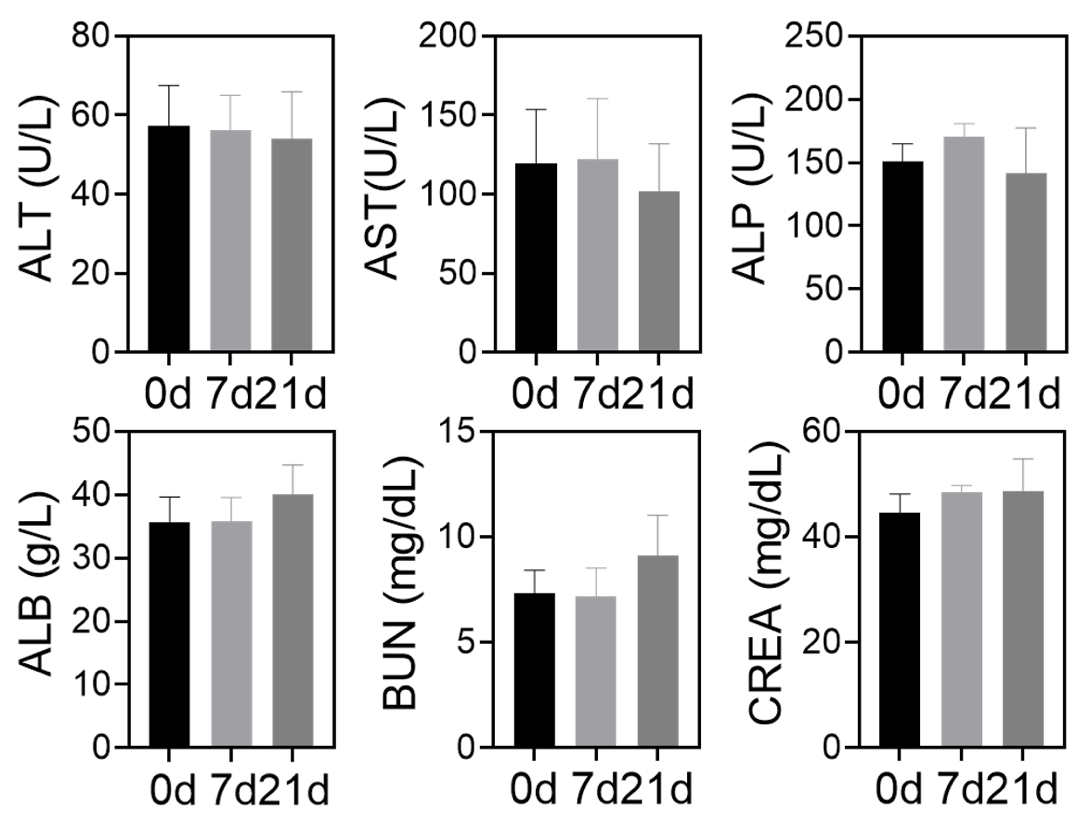


**Figure S16.** Biochemical analysis of BALB/c mice on day 0, 7 and 21 after intravenous injection of PBS or PMIA. Data are presented as mean ± SD (n = 7/group).


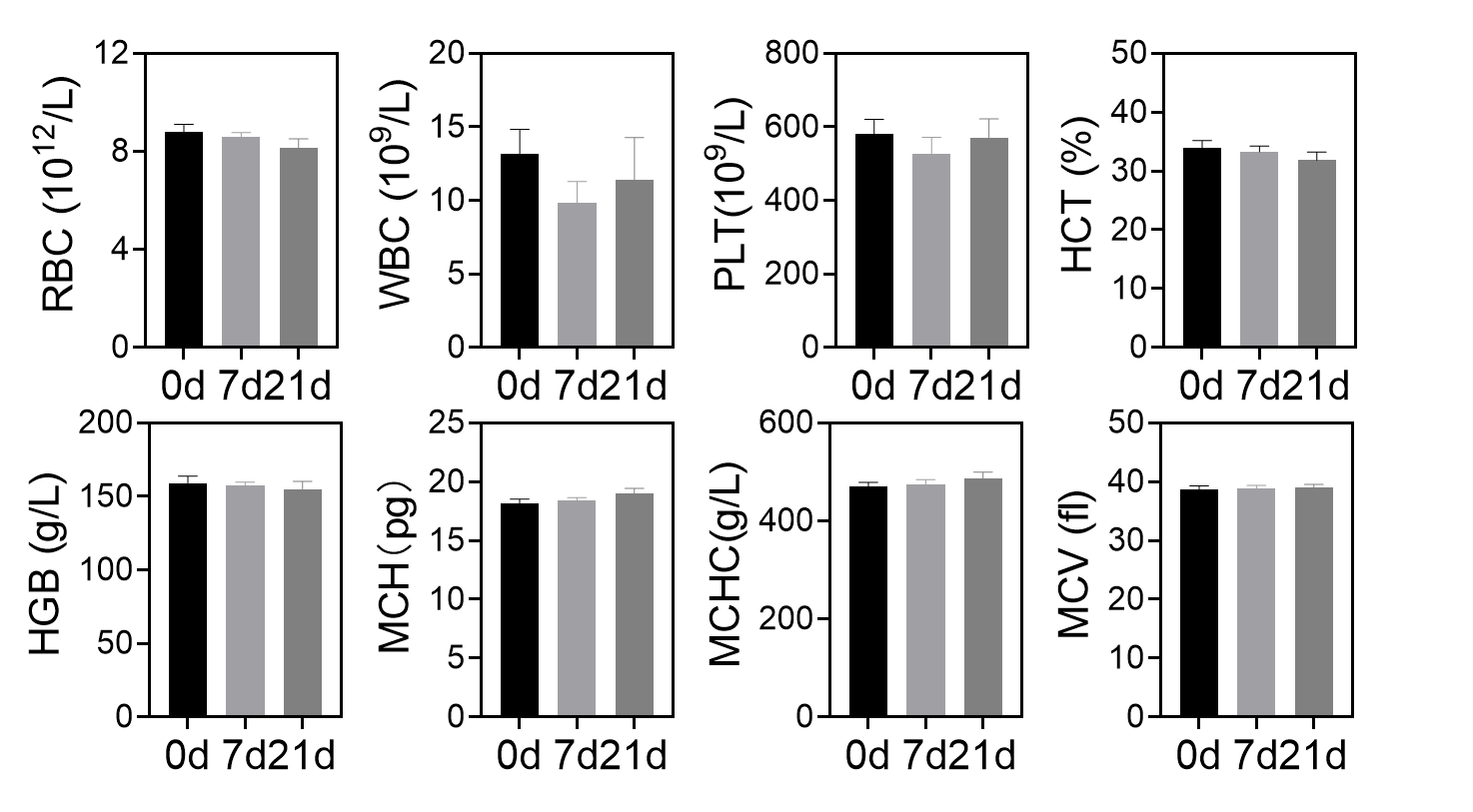


**Figure S17.** Blood analysis of BALB/c mice on day 0, 7 and 21 after intravenous injection of PBS or PMIA. Data are presented as mean ± SD (n = 7/group).


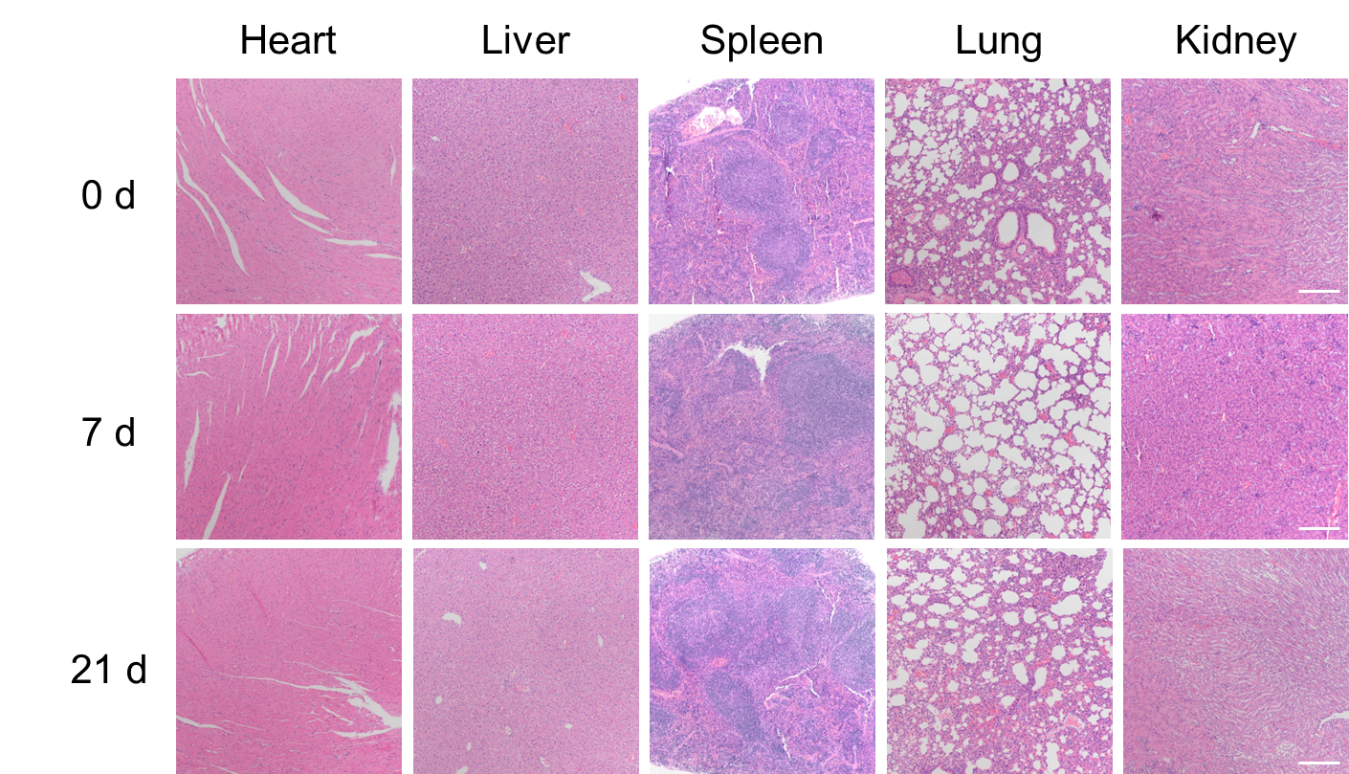


**Figure S18.** Hematoxylin and eosin (H&E) staining of main organs (heart, liver, spleen, lung, and kidney) of BALB/c mice on day 0, 7 and 21 after intravenous injection of PBS or PMIA. Scale bar, 300 µm.

**References**

[1] X. Hu, N. Wang, X. Guo, Z. Liang, H. Sun, H. Liao, F. Xia, Y. Guan, J. Lee, D. Ling, F. Li, *Nano-Micro Lett.* **2022**, 14, 101.

[2] N. Wang, P. Li, J. Zhao, Y. Liu, X. Hu, D. Ling, F. Li, *Nano Today* **2023**, 50, 101827.

[3] X. Guo, H. Liao, J. Tian, C. Yang, F. Xia, W. Liang, N. Wang, P. Li, B. Zhang, L. Gong, X. Hu, L. Zhang, F. Li, D. Ling, *Cell Rep. Phys. Sci.* **2021**, 2, 100538.

[4] A. F. Oskooi, D. Roundy, M. Ibanescu, P. Bermel, J. D. Joannopoulos, S. G. Johnson, *Comput. Phys. Commun.* **2010**, 181, 687.

[5] S. Babar, J. H. Weaver, *Appl. Opt.* **2015**, 54, 477.

[6] E. D. Palik, *Handbook of optical constants of solids*, Academic Press Boston, **1985**.
